# Solution structures of human myeloma IgG3 antibody reveal extended Fab and Fc regions relative to the other IgG subclasses

**DOI:** 10.1016/j.jbc.2021.100995

**Published:** 2021-07-22

**Authors:** Valentina A. Spiteri, Margaret Goodall, James Doutch, Robert P. Rambo, Jayesh Gor, Stephen J. Perkins

**Affiliations:** 1 Department of Structural and Molecular Biology, Division of Biosciences, University College London, London, United Kingdom; 2 Institute for Biomedical Research, University of Birmingham, Birmingham, United Kingdom; 3 ISIS Facility, STFC Rutherford Appleton Laboratory, Harwell Campus, Didcot, Oxfordshire, United Kingdom; 4 Diamond Light Source Ltd, Harwell Science and Innovation Campus, Didcot, Oxfordshire, United Kingdom

**Keywords:** analytical ultracentrifugation, antibody modeling, small-angle neutron scattering, human IgG subclasses, small angle X-ray scattering, AUC, analytical ultracentrifugation, IgG3, immunoglobulin G subclass 3, MD, molecular dynamics, PCA, principal component analyses, PDB, Protein Data Bank, PNGase F, peptide:N-glycosidase F, *R*_*G*_, radius of gyration, SANS, small-angle neutron scattering, SAXS, small-angle X-ray scattering, TAMC, Torsion Angle Monte Carlo

## Abstract

Human immunoglobulin G subclass 3 (IgG3) possesses a uniquely long hinge region that separates its Fab antigen-binding and Fc receptor-binding regions. Owing to this hinge length, the molecular structure of full-length IgG3 remains elusive, and the role of the two conserved Fc glycosylation sites are unknown. To address these issues, we subjected glycosylated and deglycosylated human myeloma IgG3 to multidisciplinary solution structure studies. Using analytical ultracentrifugation, the elongated structure of IgG3 was determined from the reduced sedimentation coefficients *s*^*0*^_*20,w*_ of 5.82 to 6.29 S for both glycosylated and deglycosylated IgG3. X-ray and neutron scattering showed that the Guinier *R*_*G*_ values were 6.95 nm for glycosylated IgG3 and were unchanged after deglycosylation, again indicating an elongated structure. The distance distribution function *P(r)* showed a maximum length of 25 to 28 nm and three distinct maxima. The molecular structure of IgG3 was determined using atomistic modeling based on molecular dynamics simulations of the IgG3 hinge and Monte Carlo simulations to identify physically realistic arrangements of the Fab and Fc regions. This resulted in libraries containing 135,135 and 73,905 glycosylated and deglycosylated IgG3 structures, respectively. Comparisons with the X-ray and neutron scattering curves gave 100 best-fit models for each form of IgG3 that accounted for the experimental scattering curves. These models revealed the first molecular structures for full-length IgG3. The structures exhibited relatively restricted Fab and Fc conformations joined by an extended semirigid hinge, which explains the potent effector functions of IgG3 relative to the other subclasses IgG1, IgG2, and IgG4.

Immunoglobulin IgG3 is one of the four subclasses IgG1 to IgG4 that make up the human IgG antibodies. It is the third most abundant IgG in serum and comprises 5% to 8% of serum IgG. IgG3 plays a role in protection against intracellular bacteria, parasites, and viruses. IgG3 is unique in the four subclasses, as it is the only subclass that has not as yet been exploited as a biotherapeutic. It is of particular interest because of its elongated hinge with 62 amino acids and 11 disulfide bonds that connects the Fab and Fc regions (Fig. 1*A*), which potentially gives the Fab and Fc regions in IgG3 a greater rotational freedom than in IgG1, IgG2, and IgG4, which have shorter hinges of lengths 15, 12, and 12 residues, respectively (1). IgG3 mediates a broad range of effector functions, including being the most effective activator of complement through the binding of C1q and able to initiate antibody effector functions such as antibody-dependent cell-mediated cytotoxicity. The superior ability of IgG3 to bind to FcγR receptors and C1q was thought to be due to its elongated hinge (2); however, this can also be attributed to variation in the IgG3 sequence in the C_H_2 region (3). Of all the IgG subclasses, IgG3 has the most known allotypes, with about 15 in total that correspond to polymorphisms in its constant regions that vary the hinge length or the Fc sequence (3). Some IgG3 allotypes have a reduced half-life of about 7 days, compared with the longer 21-day half-life for IgG1, IgG2, and IgG4 (1). This reduced half-life is attributed to a His435Arg substitution in the Fc region (EU numbering) (4). This His435Arg substitution is also critical as it blocks IgG3 from binding to a protein A column, thus allowing for IgG3 to be purified from IgG1, IgG2, and IgG4 in human serum (5).

**Figure 1:**
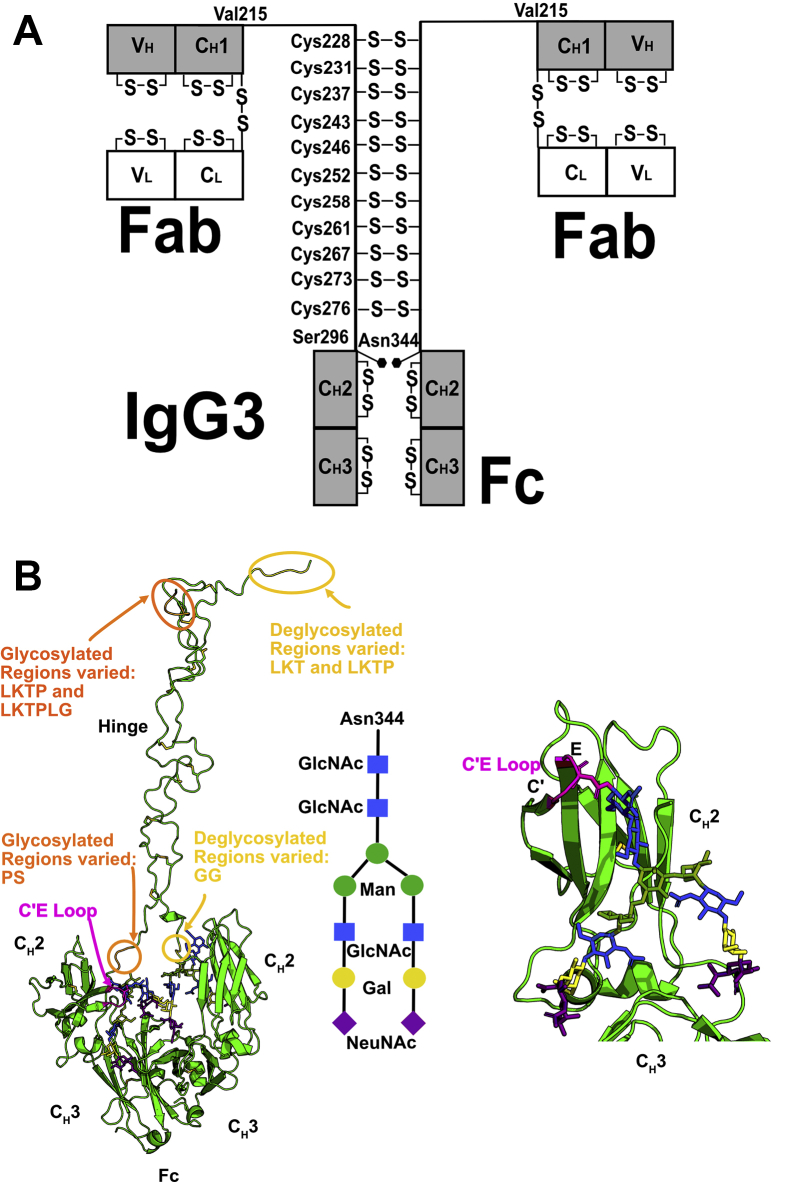
**The human IgG3 domains and its glycosylation.***A*, the heavy chains comprise V_H_, C_H_1, C_H_2, and C_H_3 domains, and the light chains comprise V_L_ and C_L_ domains. The heavy chains are connected in the hinge region by 11 Cys-Cys disulfide bridges at Cys228, Cys231, Cys237, Cys243, Cys246, Cys252, Cys258, Cys261, Cys267, Cys273, and Cys276 as shown. An N-linked oligosaccharide at Asn297 (EU numbering) or Asn344 (continuous numbering) is present on each of the C_H_2 domains. The hinge region connecting the Fab and Fc regions was constructed from 70 residues ^216^ELKTPLGDTTHTCPRCP(EPKSCDTPPPCPRCP)_3_APELLGGP^285^. *B*, at the *left*, the IgG3 Fc glycans at both Asn297 (Asn344) residues in the Fc region are shown as stick models. The three hinge peptides that were conformationally varied in the TAMC searches for glycosylated models are shown in *orange*. Those for the deglycosylated models are shown in *yellow*. The central schematic shows the glycosylation pattern used in this study. At the *right*, the detailed view of a single C_H_2 domain with its glycan chain is shown, with the glycan colors coordinated with those in the schematic. Gal, galactose; GlcNAc, N-acetyl glucosamine; Man, mannose; NeuNAc, N-acetyl neuraminic acid

The molecular structure of full-length human IgG3 is poorly understood, given that its elongated hinge has made structural studies difficult using methods such as X-ray crystallography. Nonetheless a high-resolution structure for the IgG3-Fc fragment (Protein Data Bank [PDB] ID: 5W38) (6) is available. Several solution studies of full-length IgG3 using small-angle X-ray scattering (SAXS) and analytical ultracentrifugation (AUC) have been reported (7, 8, 9). These studies showed that IgG3 has an elongated structure compared with IgG1, IgG2, and IgG4. By SAXS, IgG3 was found to have a more flexible hinge than IgG1 and IgG2 (7). Structural predictions of the hinge have suggested that its length should be around 9 nm (10). IgG3 also showed partial O-glycosylation at the IgG3 hinge region (11). All four human IgG subclasses have a conserved N-linked glycosylation site in the Fc region, which plays a key functional role (Fig. 1*B*). A complex-type biantennary glycan with a Man_3_GlcNAc_2_ core and two NeuNAc.Gal.GlcNAc antennae is attached at Asn297 (EU numbering) on each C_H_2 domain (12) (Fig. 2). This glycan is chemically heterogenous (13) and influences the interaction with FcγR receptors and complement C1q binding (14). The structural role of the two Fc glycans in IgG3 is not well understood, but deglycosylated IgG3 was shown to have impaired binding to FcγR receptors, leading to the conclusion that FcγR binding depended on the conformation of the Fc region in IgG3 (15).

**Figure 2:**
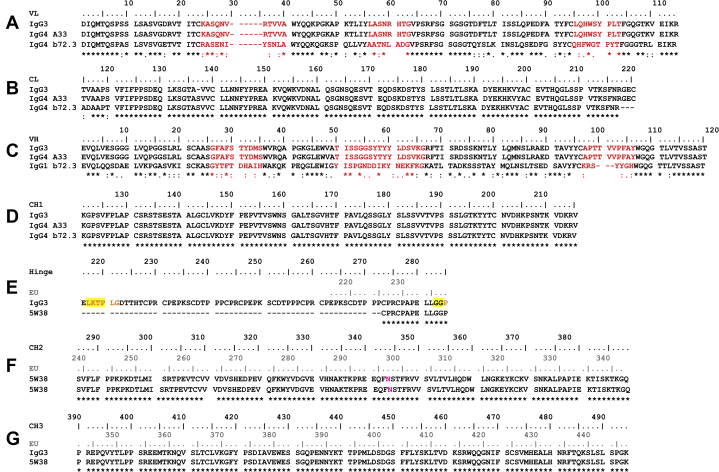
**Sequence alignment of IgG3.***A–G*, given that no IgG3 sequence was available, the IgG3 Fab sequence was taken to be that from IgG4 A33 Fab (Experimental procedures). Its atomistic modeling was based on the IgG4 b72.3 crystal structure (PDB ID: 1BBJ) whose sequence is included. The IgG3 hinge and Fc sequences were taken from its crystal structure (PDB ID: 5W38). Beneath the alignments, (∗) indicates full conservation, (:) indicates conservation between groups of strongly similar properties based on the Gonnet PAM 250 matrix, (.) indicates conservation between groups of weakly similar properties, and a space indicates no conservation. *A* and *B*, the V_L_ and C_L_ domains; *C–E*, the V_H_ and C_H_1 domains and the hinge, with the TAMC-varied peptides for glycosylated IgG3 shown in *orange* and for deglycosylated IgG3 shown in *yellow*. In *A* and *C*, the three CDR sequences are in *red*. *F, G*, the C_H_2 and C_H_3 domains, with Asn297 (Asn344) in *magenta*. In *E–G*, the standard EU residue numbering that matches IgG1, IgG2, and IgG4 is shown in *gray*, and the continuous numbering is shown in *black*.

The issues of the unknown IgG3 molecular structure and a role for its glycans were here addressed by a joint application of AUC, SAXS, small-angle neutron scattering (SANS), and atomistic modeling to myeloma human IgG3. This powerful solution structural strategy has recently provided molecular structures for glycosylated and deglycosylated monoclonal IgG1, and also human myeloma IgG2 (16, 17). AUC provides overall solution structures for IgG3 as well as confirms its monodispersity in solution. Scattering provides low-resolution structural data on IgG3. Here, SAXS provides datasets measured in high positive solute–solvent contrast, in which the contribution of the hydrophilic surface regions of the glycoprotein are accentuated, whereas SANS measured with heavy water buffers provides datasets measured in high negative solute–solvent contrast, in which the contribution of the buried hydrophobic core of the glycoprotein is accentuated (18, 19, 20). The tightly bound hydration layer is detected by SAXS because its electron density is similar to that of the protein and not to bulk water, whereas this same hydration layer is almost invisible by SANS measured in heavy water, because its nuclear density is almost the same as that of bulk water. The reproducibility of the SAXS and SANS datasets confirms the outcome of both methods, because experimental artefacts from radiation effects in SAXS and aggregation in heavy water by SANS may perturb the outputs of either method. SAXS and SANS datasets can now be represented as molecular structures by the relatively recent development of atomistic modeling using molecular dynamics and Monte Carlo methods to fit the scattering curves (21). Here, we apply this joint AUC-SAXS-SANS approach to determine experimentally the first full-length IgG3 molecular structure for myeloma IgG3 in order to explain better its immune function. We also discuss the effect of the removal of the Fc glycans on the molecular structure of full-length IgG3.

## Results

### Purification and characterization of glycosylated and deglycosylated IgG3

**Figure 3:**
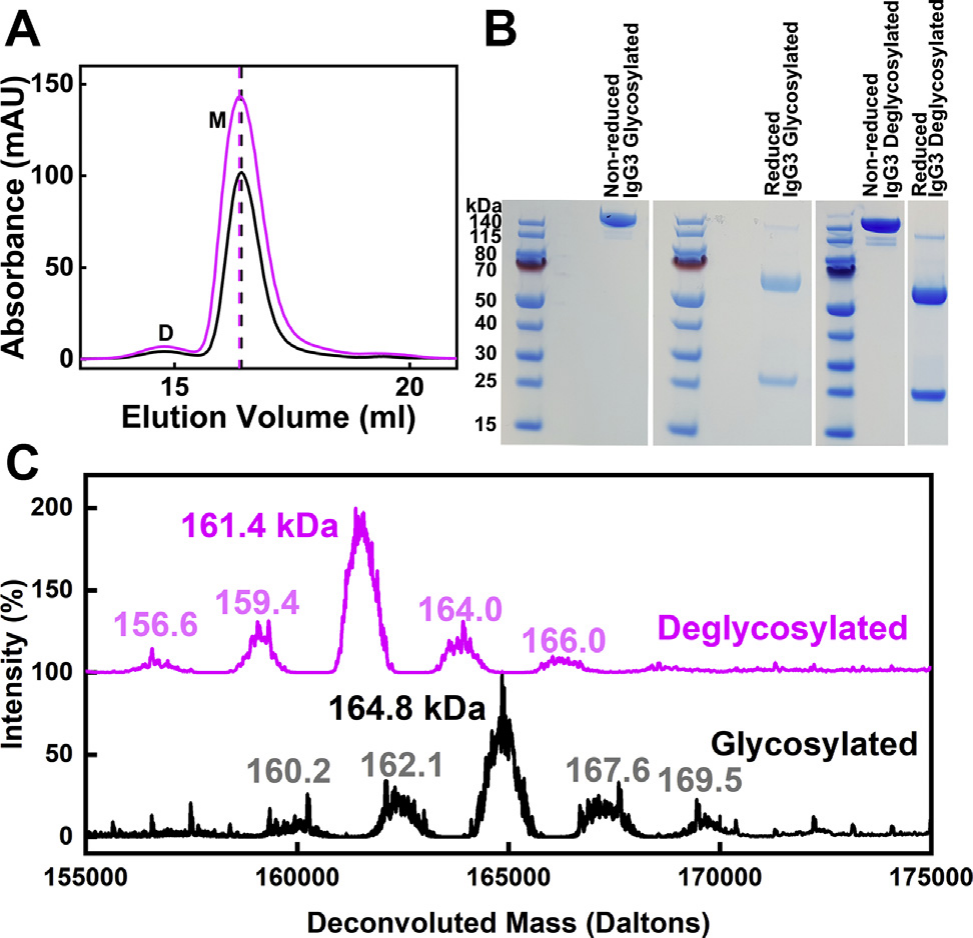
**Purification, SDS-PAGE, and mass spectrometry of human glycosylated and deglycosylated IgG3.***A*, elution peaks from a Superose 6 Increase 10/300 gel filtration column for a glycosylated IgG3 sample (*black*) and a deglycosylated IgG3 sample (*magenta*). The *dashed vertical lines* indicate the peak positions. *B*, lanes 1, 3, and 5, molecular mass markers are denoted in kDa. Lanes 2 and 4, nonreduced and reduced SDS-PAGE of glycosylated IgG3 after gel filtration. Lanes 6 and 7, reduced and nonreduced SDS-PAGE of deglycosylated IgG3 after gel filtration. *C*, mass spectra of glycosylated (*black*) and deglycosylated (*magenta*) IgG3.
(ii) When the IgG3 samples were submitted to nonreducing and reducing SDS-PAGE analyses at equimolar concentrations, purified glycosylated and deglycosylated IgG3 showed a single band above 140 kDa on 4% to 12% Bis Tris NuPage gel under nonreducing conditions, which is consistent with the assumed mass of ∼158 kDa for IgG3 (Fig. 3*B*). Under reducing conditions, two bands were present corresponding to the heavy chain (with an apparent mass of ∼50–70 kDa) and the light chain (with an apparent molecular mass of ∼25 kDa) (Fig. 3*B*). These apparent molecular masses were as expected from the known sequence (Fig. 2).
(iii) Liquid chromatography mass spectrometry measurements showed a major peak and four minor peaks for glycosylated (in black) and deglycosylated (in magenta) IgG3 in a mass range of 160.2 to 169.5 kDa for glycosylated IgG3 and 156.6 to 166.0 kDa for deglycosylated IgG3 (Fig. 3*C*). These peaks were unaffected by longer PNGase F digestion times. The existence of five peaks is attributed to up to as many as six weakly occupied O-linked glycans of sizes 1.9 to 2.8 kDa in the IgG3 hinge (11). The impact of these O-linked glycans, if present, on the scattering data below is expected to be minimal because of their central location in the IgG3 structure and their low abundance and were not characterized further. The most intense IgG3 glycosylated population had an observed deconvoluted mass of 164,847 Da. For deglycosylated IgG3 (in magenta) the most intense population had an observed deconvoluted mass at 161,383 Da. The mass of each N-glycan chain was calculated by subtracting the glycosylated and deglycosylated masses and halving the outcome to give 1732 Da. This value agrees well with an assumed N-glycan composition of Gal_2_Man_3_GlcNAc_4_ that gives a mass of 1622 Da.

Human IgG3 was purified in high yields from human myeloma serum (Experimental procedures). In order to work with IgG3 with and without glycans, the deglycosylation of myeloma IgG3 was set up using peptide:N-glycosidase F (PNGase F) digests according to the manufacturer's protocol (Experimental procedures). The completeness of deglycosylation was verified by a combination of size-exclusion gel filtration, SDS-PAGE, and mass spectrometry, and also by analytical ultracentrifugation (see below):

(i) Deglycosylation of IgG3 was completed after 10 h. The elution of the IgG3 PNGase F-treated product from a gel filtration column slightly preceded that for native glycosylated IgG3 (Fig. 3*A*). Both forms eluted as a main symmetrical peak at 16.43 and 16.38 ml for glycosylated and deglycosylated IgG3, respectively (Fig. 3*A*). Trace amounts of a possible dimer just before 15 ml were discarded. This process ensured that the IgG3 sample was monodisperse with no aggregates present immediately before analytical ultracentrifugation or scattering experiments.

### Analytical ultracentrifugation of glycosylated and deglycosylated IgG3

**Figure 4:**
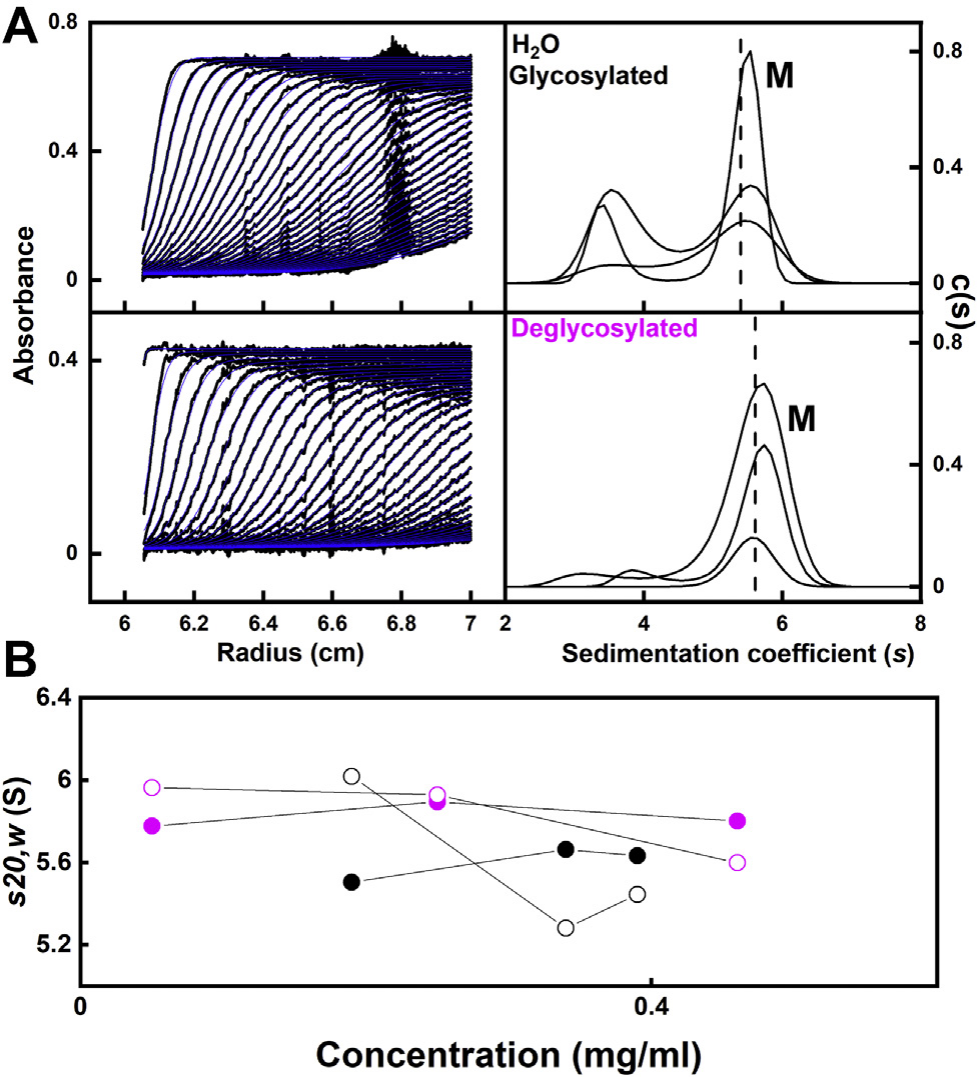
**Sedimentation velocity analyses of glycosylated and deglycosylated IgG3.***A*, the experimentally observed sedimentation boundaries using Absorbance optics for a concentration series of glycosylated IgG3 and deglycosylated IgG3 in histidine buffer in light water. Scans were recorded at 30,000 rpm and 20 °C, from which 31 to 60 boundaries (*black outlines*) are shown from totals of up to 896 scans. The SEDFIT fits are shown in *blue*. The peaks in the corresponding size distribution analyses *c(s)* revealed a monomer peak (M) at *s*_*20,w*_ values of 5.78 to 6.33 S for glycosylated and deglycosylation IgG3 in light water. *B*, the *s*_*20,w*_ values for the monomer peaks are shown as a function of concentration for glycosylated (●) and deglycosylated () IgG3. The interference data analyses are denoted by *open symbols*.

Sedimentation velocity experiments characterized the masses and solution structures of glycosylated and deglycosylated IgG3. The SEDFIT analyses of the boundaries involved fits of as many as 600 scans, and the good agreement between the experimental boundary scans and fitted lines is clear (left, Fig. 4*A*). From the *c(s)* analyses, the molecular masses of the IgG3 monomer peak were 108 (glycosylated) and 122 kDa (deglycosylated) in light water. In heavy water the molecular masses of the IgG3 monomer peak were 139 (glycosylated) and 156 kDa for deglycosylated. These values were comparable with the composition-calculated masses of 158 and 154 kDa for the glycosylated and deglycosylated IgG3 monomers, respectively. These also agree well with the values from mass spectrometry of 165 and 161 kDa for glycosylated and deglycosylated IgG3, respectively (Fig. 3*C*). In the resulting size distribution analyses *c(s)*, a clear monomer peak that monitored the overall IgG3 solution structure was observed at average *s*_*20,w*_ values of 5.82 ± 0.06 S for glycosylated IgG3 and 6.29 ± 0.05 S for deglycosylated IgG3 in light water (Fig. 4*A*; Table 1). Because the samples were dilute, the expected 0.2 S reduction (3%) in the *s*_*20,w*_ values after deglycosylation to follow the 4 kDa reduction (3%) in the IgG3 mass was not seen because of noisy data at low concentrations. These *s*_*20,w*_ values were consistent with previous AUC studies of glycosylated IgG3 that reported *s*_*20,w*_ values of 6.11 ± 0.03 S (8) and 5.90 S ± 0.02 (9). In heavy water, monomer and no dimers were also seen by AUC (data not shown). The calculation presumes that the IgG3 conformation (*i.e.*, the frictional coefficient) is unchanged after deglycosylation. Variable minor peaks at 3 to 4 S were seen adjacent to the main monomer peak (M) (Fig. 4*A*); they were attributable to minor fragmentation of IgG3 and/or artefacts caused by small sample–buffer optical mismatches. In conclusion, given that no concentration dependences were seen in Figure 4*B*, these datasets showed that IgG3 was monomeric in solution, as required for scattering studies.

**Table 1:** Experimental data by X-ray and neutron scattering and analytical ultracentrifugation for glycosylated and deglycosylated IgG3

Small angle scattering data	Concentration (mg/ml)	*R*_*G*_ (nm)	*R*_*XS-1*_ (nm)	*R*_*XS-2*_ (nm)	*L* (nm)
X-ray data					
IgG3 glycosylated	2.46	6.98 ± 0.62	1.41 ± 0.17	1.60 ± 0.10	28
	2.00	6.99 ± 0.63	1.41 ± 0.18	1.61 ± 0.10	28
	1.50	6.96 ± 0.67	1.41 ± 0.15	1.61 ± 0.22	28
	1.00	6.99 ± 0.82	1.41 ± 0.19	1.60 ± 0.27	25
	0.50	6.84 ± 0.90	1.32 ± 0.27	1.61 ± 0.31	28
IgG3 deglycosylated	1.00	7.05 ± 0.71	1.41 ± 0.19	1.59 ± 0.15	28
	0.50	6.98 ± 1.06	1.41 ± 0.29	1.58 ± 0.25	28
Neutron data					
IgG3 glycosylated	1.85	6.26 ± 1.07	0.89 ± 0.30	1.15 ± 0.21	28
	0.70	6.20 ± 1.33		0.97 ± 0.24	25
IgG3 deglycosylated	1.21	6.24 ± 1.58	1.10 ± 0.35	1.22 ± 0.27	25
	0.65	6.33 ± 1.64		1.02 ± 0.43	27


AUC data	Concentration (mg/ml)	*s*_*20,w*_ (S)
IgG3 glycosylated in light water	0.39	5.80
	0.34	5.90
	0.19	5.78
IgG3 deglycosylated in light water	0.46	6.24
	0.25	6.29
	0.05	6.33

### X-ray and neutron scattering of glycosylated and deglycosylated IgG3

The solution structures of glycosylated and deglycosylated IgG3 samples were characterized by X-ray and neutron scattering. The two methods provided different perspectives of the same solution structure. X-rays in light water buffers detect the hydration shell surrounding the protein structure, whereas neutrons in heavy water buffers see a much reduced effect of this hydration shell because of the different solute–solvent contrast in use (18, 19, 20).

**Figure 5:**
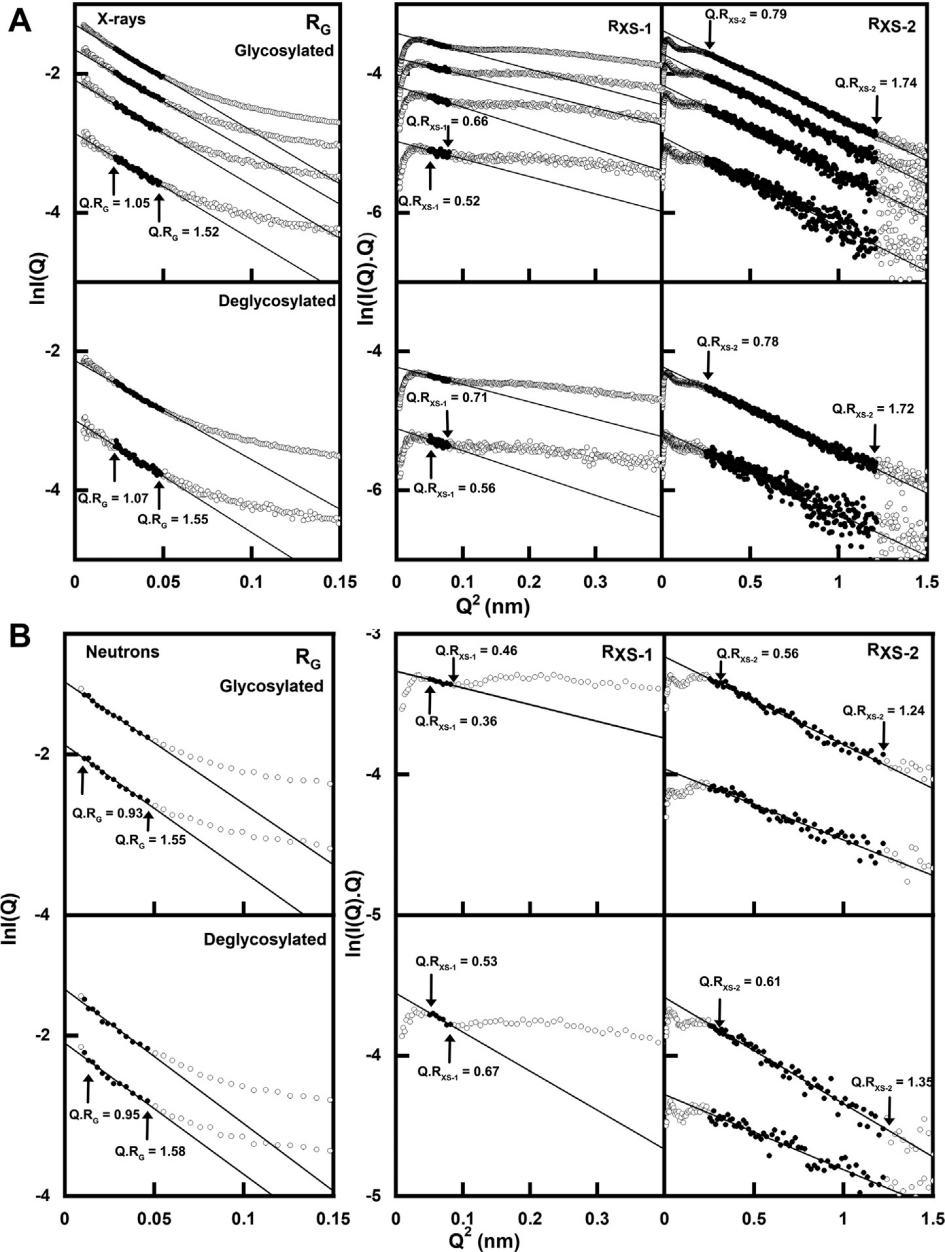
**X-ray and neutron Guinier *R***_***G***_**and *R***_***XS***_**analyses for glycosylated and deglycosylated IgG3.***A*, the SAXS curves for glycosylated and deglycosylated IgG3 at concentrations of 0.50 to 2.46 mg/ml. The *filled circles* between the arrows represent the *Q·R*_*G*_ and *Q·R*_*XS*_ fit ranges used to determine the *R*_*G*_ and *R*_*XS*_ values. The *Q* range used for the *R*_*G*_ values was 0.10 to 0.22 nm^−1^; those for the *R*_*XS-1*_ and *R*_*XS-2*_ values were 0.22 to 0.28 nm^−1^ and 0.50 to 1.10 nm^−1^, respectively. *B*, the SANS curves for glycosylated and deglycosylated IgG3 at concentrations of 0.65 to 1.85 mg/ml. The *Q* range used for the *R*_*G*_ values was 0.10 to 0.22 nm^−1^ and those for the *R*_*XS-1*_ and *R*_*XS-2*_ values were 0.23 to 0.28 nm^−1^ and 0.50 to 1.10 nm^−1^, respectively.

**Figure 6:**
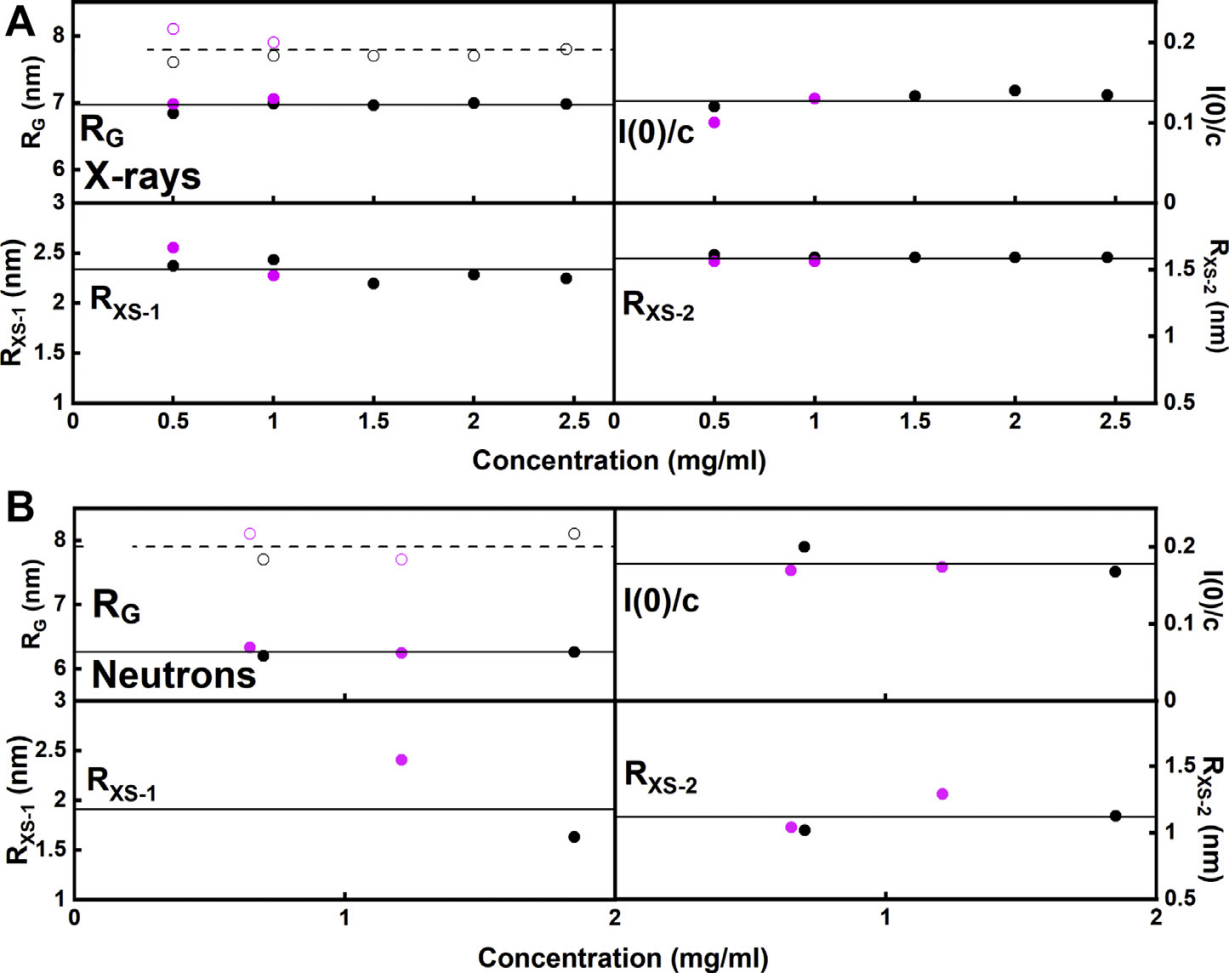
**Concentration dependence of the SAXS and SANS Guinier analyses.** The *filled symbols* show the values determined from the Guinier analyses and the open symbols in the *R*_*G*_ panels indicate those determined from the *P(r)* analyses. The colors denote the glycosylated (*black*) and deglycosylated (*magenta*) IgG3. *A*, the SAXS *R*_*G*_, *I(0)/c*, *R*_*XS-1*_, and *R*_*XS-2*_ values for glycosylated (●, ○) and deglycosylated (, ) IgG3. The *solid lines* corresponded to the mean *R*_*G*_ values determined from the Guinier analyses for glycosylated and deglycosylated IgG3, and the *dashed lines* to the mean *R*_*G*_ values determined by *P(r)* analyses. *B*, the SANS *R*_*G*_, *I(0)/c*, *R*_*XS-1*_, and *R*_*XS-2*_ values for glycosylated and deglycosylated IgG3, each corresponding to a single measurement in histidine buffer in ^2^H_2_O. The *solid* and *dashed lines* correspond to the mean values for glycosylated and deglycosylated IgG3.

The X-ray data collection at IgG3 concentrations between 0.5 and 2.46 mg/ml used time frame analyses to ensure the absence of radiation damage effects. The resulting *R*_*G*_ and *R*_*XS-1*_/*R*_*XS-2*_ values monitor the elongation of the overall IgG3 structure and its approximate cross-sectional structures, respectively. Guinier analyses resulted in high-quality linear plots for all samples and revealed three distinctive regions of the *I(Q)* curves, which is seen in the scattering curves for antibodies (22, 23, 24). From these, the *R*_*G*_, *R*_*XS-1*_, and *R*_*XS-2*_ values from the individual scattering curves were obtained within satisfactory *Q·R*_*G*_ and *Q·R*_*XS*_ limits of 1.05 to 1.55, 0.52 to 0.71, and 0.78 to 1.74, respectively (Fig. 5*A*). The mean X-ray *R*_*G*_ values that monitor the overall structure for glycosylated and deglycosylated IgG3 samples were similar, being 6.95 ± 0.06 and 7.02 ± 0.05 nm, respectively (Table 1). The *R*_*G*_ value for glycosylated IgG3 compared well with previous SAXS studies, which reported *R*_*G*_ values of 6.93 (7), 6.20 (9), and 7.16 nm (8). The *R*_*XS-1*_ values from the individual curves (Fig. 6*A*) is an approximate monitor of the cross-sectional Fab and Fc arrangement in glycosylated IgG3 and deglycosylated IgG3. The mean X-ray *R*_*XS-1*_ values for glycosylated and deglycosylated IgG3 were both typically 1.41 ± 0.2 nm, showing that the spatial arrangement of the Fab and Fc regions was unchanged following glycan removal. These values were notably reduced from *R*_*XS-1*_ values of 2.5 (±0.1) nm measured for monoclonal IgG1 before and after deglycosylation (17). This difference is attributed to the effect of the long hinge region in IgG3 that separated the Fab and Fc regions. This also had the effect of reducing the *Q* range that could be used for the *R*_*XS-1*_ fits compared with IgG1. The *R*_*XS-2*_ values are an approximate monitor of the mean cross-sectional dimensions of the individual Fab and Fc regions. The mean *R*_*XS-2*_ values for glycosylated and deglycosylated IgG3 were similar in a range of 1.59 to 1.61 (±0.2) nm. This indicates that the averaged cross-sections of the two Fab and one Fc region were unchanged before and after deglycosylation. Similar *R*_*XS-2*_ values of 1.4 (±0.1) nm were recently reported for monoclonal IgG1 before and after deglycosylation (17). No concentration dependences were seen for IgG3, this being seen from the Guinier values that remained unchanged within error (Fig. 6). These results showed that the overall and individual Fab and Fc regions were unchanged in their solution structures between 0.5 and 2.5 mg/ml.

The corresponding neutron scattering datasets for glycosylated and deglycosylated IgG3 in 100% ^2^H_2_O buffer were acquired at similar concentrations of 0.65 to 1.85 mg/ml. Again, the Guinier analyses revealed high-quality linear fits for the *R*_*G*_, *R*_*XS-1*_, and *R*_*XS-2*_ parameters (Fig. 5*B*). A concentration dependence was not observed for IgG3, this being seen from the *I(0)/c* values that remained unchanged within error (Fig. 6*B*). This difference between the neutron and X-ray datasets is attributable to the fewer data points obtained with neutrons, leading to reduced precision in the datasets. The mean neutron *R*_*G*_ values for glycosylated and deglycosylated IgG3 were similar at 6.23 ± 0.04 and 6.29 ± 0.04 nm, respectively (Fig. 6*B*; Table 1). The mean neutron *R*_*XS-1*_ values for glycosylated and deglycosylated IgG3 were similar at 0.80 ± 0.3 and 1.05 ± 0.3 nm, respectively (Table 1). The mean neutron *R*_*XS-2*_ values for glycosylated and deglycosylated IgG3 were unchanged at 1.06 ± 0.2 and 1.12 ± 0.3 nm, respectively. Overall, the neutron *R*_*G*_ values confirmed the X-ray analyses; however, the neutron *R*_*XS-1*_ and *R*_*XS-2*_ are reduced compared with their X-ray values, this being attributed to the reduced contribution of the hydration shell to these measurements.

**Figure 7:**
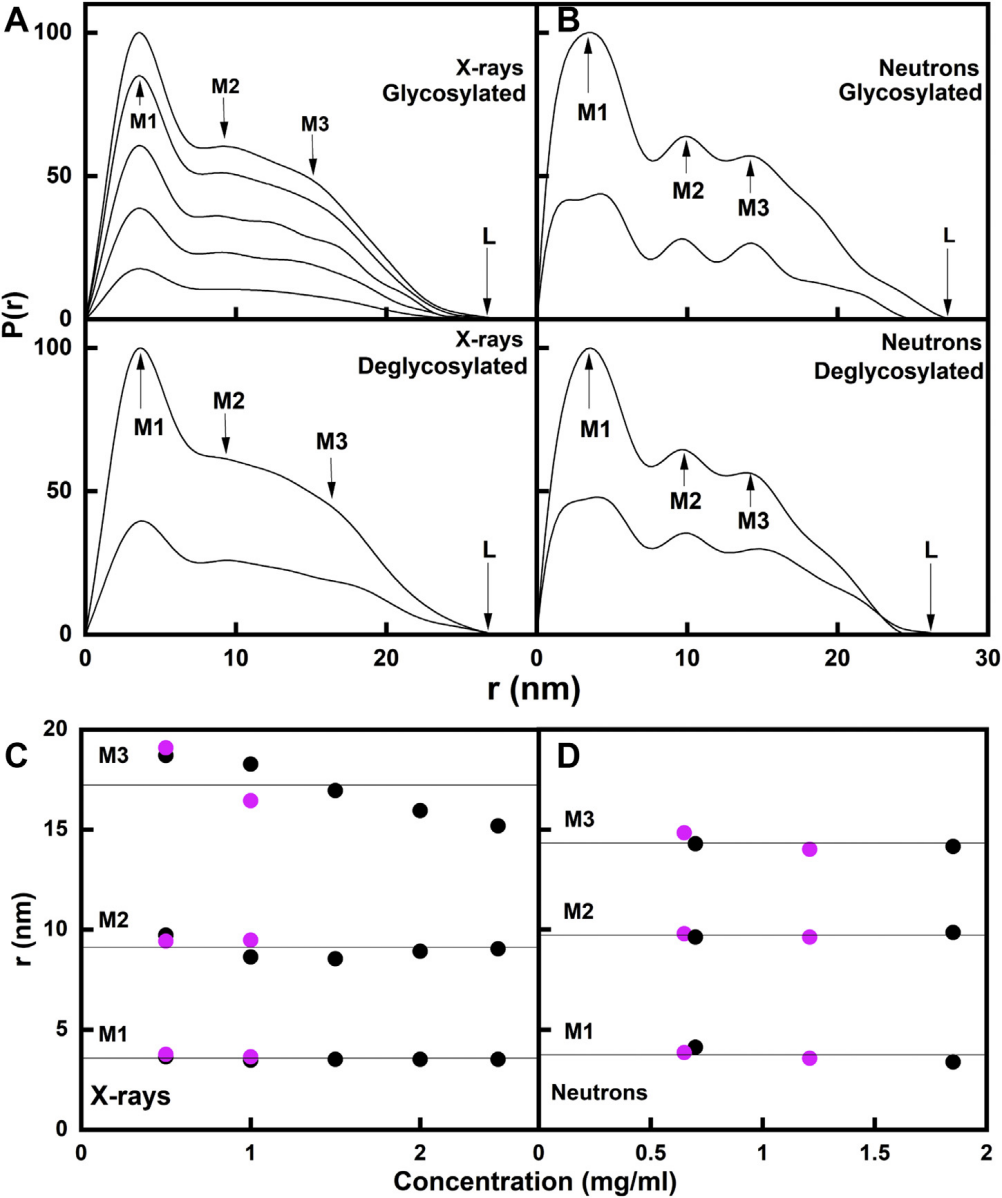
**SAXS and SANS distance distribution analyses *P(r)* for each of glycosylated and deglycosylated IgG3.** Glycosylated and deglycosylated IgG3 are denoted in *black* and *magenta*. *A*, the peak maxima at *M1, M2*, and *M3* and the maximum length *L* are indicated by *arrows*. The SAXS and SANS *P(r)* curves for glycosylated and deglycosylated IgG3 are shown at concentrations of 0.50 to 2.46 mg/ml. *B*, the corresponding *P(r)* curves for the SANS curves for IgG3 at concentrations of 0.65 to 1.85 mg/ml. *C* and *D*, the concentration dependence of the *M1*, *M2*, and *M3* peaks for glycosylated (•) and deglycosylated () IgG3 are shown. The *fitted lines* are the mean values for glycosylated and deglycosylated IgG3 averaged together (*solid line*).

The distance distribution function *P(r)* is derived from Fourier transformation of the scattering curve *I(Q)* and provides structural information in real space on glycosylated and deglycosylated IgG3. The X-ray and neutron *P(r)* analyses gave *R*_*G*_ values that were similar to those from the X-ray Guinier analyses, showing that the two analyses were self-consistent (open symbols, Fig. 6). The X-ray maximum lengths *L* of glycosylated and deglycosylated IgG3 were determined from the value of *r* when the *P(r)* curve intersects zero on the *r* axis and were generally 28 nm (Fig. 7*A*). The neutron maximum lengths *L* of glycosylated and deglycosylated IgG3 were 25 to 28 nm. These were slightly smaller when compared with the X-ray value of 28 nm (Fig. 7*B*). These reductions in the neutron *R*_*G*_ and *L* values compared with the X-ray values have been previously seen in our earlier joint SAXS and SANS studies of antibodies (23). From previous SAXS experiments of IgG3, the reported maximum lengths of glycosylated IgG3 molecule were 19.5 (8) and 27.8 nm (7); the latter value agrees well with our current values (Table 1).

The maxima in the *P(r)* curves corresponded to the most frequently occurring distances between scattering elements within the IgG3 structures. For the IgG3 samples, three peaks, *M1, M2*, and *M3*, were seen (Fig. 7, *A* and *B*). Two peaks, *M1* and *M2*, are characteristic for IgG1, IgG2, and IgG4 antibodies (16, 17, 22, 23). The appearance of the third peak, *M3*, is attributed to the elongated IgG3 hinge structure and was also seen previously (7). In IgG3 the *M1* peak corresponds primarily to the shorter distances within each Fab and Fc region, and its position is expected to be almost invariant for this reason. In IgG3, *M2* now corresponds to the distances between pairs of Fab regions, whereas *M3* corresponds primarily to the longer distances between pairs of Fab and Fc regions (Figs. 1 and 7). No differences in maxima peak positions were observed between glycosylated and deglycosylated IgG3 (Fig. 7*C*). For the X-ray data, the *M1* peak was observed at 3.53 ± 0.06 nm for glycosylated IgG3 and 3.58 ± 0.09 nm for deglycosylated IgG3. The *M2* peak was observed at 8.97 ± 0.47 nm for glycosylated IgG3 and 9.11 ± 0.04 nm for deglycosylated IgG3. Although not well resolved by X-rays, the positions of the *M3* peak varied between 16 and 19 nm. The *M3* peak was observed at 17.0 ± 1.5 nm for glycosylated IgG3 and 17.2 ± 1.9 nm for deglycosylated IgG3. The neutron *P(r)* analyses reflected the same trends as the X-ray data (Fig. 7*D*), although the *M3* peak was better resolved in the neutron *P(r)* curves (Fig. 7*B*). The neutron *M1* peak was seen at 3.8 ± 0.5 nm for glycosylated IgG3 and 3.7 ± 0.2 nm for deglycosylated IgG3, the *M2* peak was seen at 9.7 ± 0.2 nm for glycosylated IgG3 and 9.7 ± 0.1 nm for deglycosylated IgG3, and the *M3* peak was seen at 14.2 ± 0.1 nm for glycosylated IgG3 and 14.4 ± 0.6 nm for deglycosylated IgG3. Both the X-ray and neutron analyses were thus consistent with each other and support the premise that the two Fab regions were well separated from the Fc region in solution.

### Atomistic modeling of glycosylated and deglycosylated IgG3

**Figure 8:**
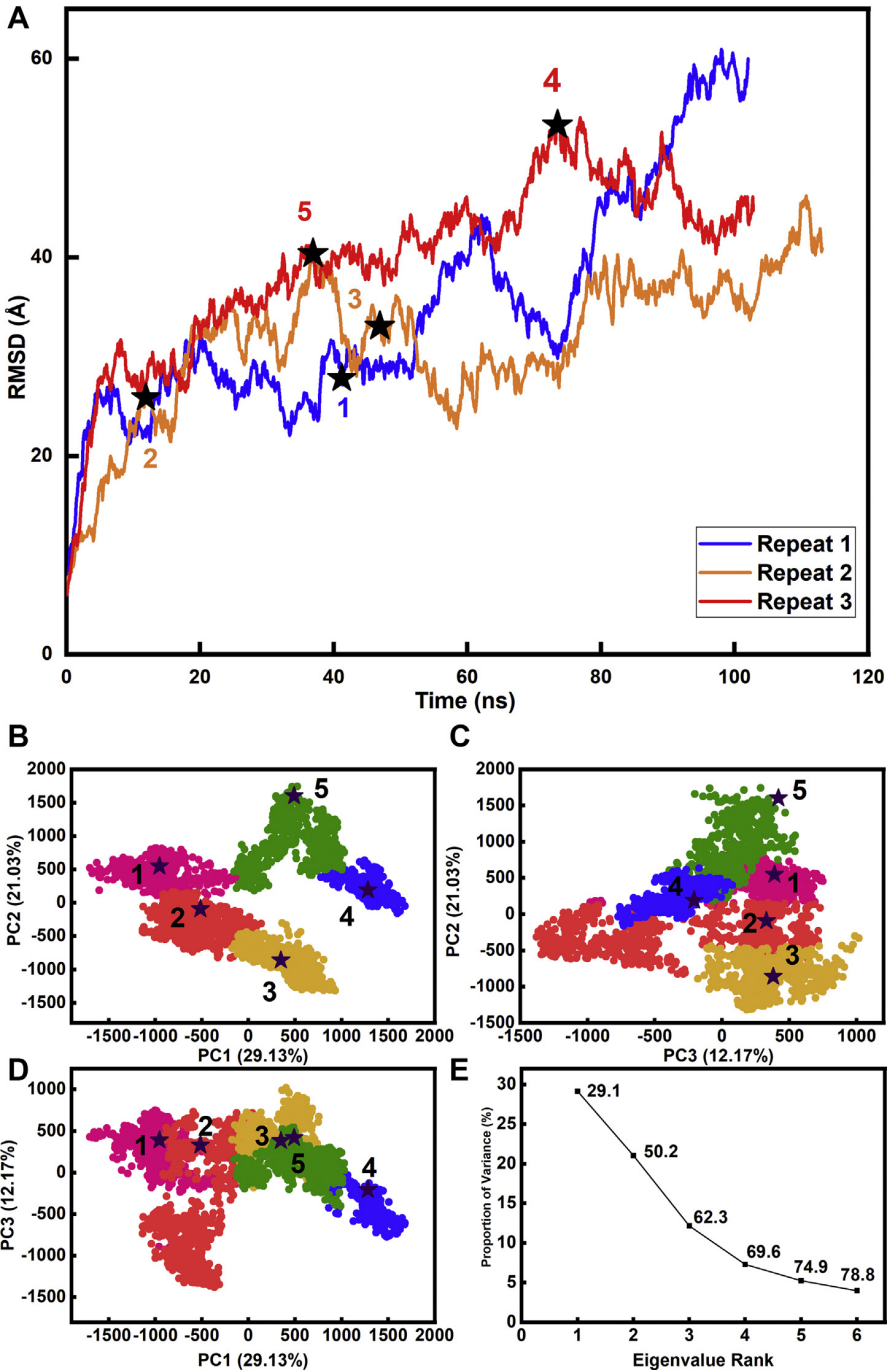
**Root-mean-square deviation (RMSD) of the hinge molecular dynamics simulations and their principal component analyses (PCA).***A*, the RMSD of the hinge residue α-carbon positions plotted against time in the molecular dynamics simulation, for repeats 1, 2, and 3 in *blue*, *red*, and *yellow*, respectively. The five numbered ★ symbols represent the midpoint for each cluster in the PCA. *B–D*, the three molecular dynamics simulations of the IgG3 hinge were combined and grouped by PCA into five groups of PC2 *versus* PC1, PC3 *versus* PC2, and PC3 *versus* PC1 (PC, principal component). The PCA groups 1, 2, 3, 4, and 5 are, respectively, colored in *magenta*, *red*, *yellow*, *green*, and *blue*. The centroid model for each cluster is represented by numbered ★ symbols. *E*, the first three eigenvalue rankings (PC1, PC2, and PC3) captured 62.3% of the variance in the molecular dynamics simulations.

Despite the observation by scattering of small but detectable changes in the scattering curves of monoclonal IgG1 following glycan removal (17), no such changes could be seen for myeloma IgG3 in the present study. The most likely reason for this is that any increase in IgG3 disorder in the Fc region would have a minimal effect on the well-separated Fab regions because of the long hinge in IgG3. The atomistic modeling simulations of the glycosylated and deglycosylated IgG3 structures were used to determine best-fit molecular structures, starting from two high-resolution crystal structures for the human Fab and Fc regions (Experimental procedures). The IgG3 sequence is that given in Figure 2, *A–G*. The Fab and Fc regions were joined by a 62-residue hinge peptide (Experimental procedures; Fig. 2*E*), built using PyMOL. This hinge was energy minimized using NAMD with the CHARMM36 forcefield to generate a relaxed structure, then the latter was simulated in triplicate using all-atom molecular dynamics in NAMD for 100 ns (Fig. 8*A*). From this, a principal component analysis (PCA) (25) on the combined simulations identified five clusters that captured the most representative hinge conformations (Fig. 8, *B–E*). Five conformations that corresponded to the mid-point of each cluster were selected for attachment to the Fab and Fc regions to give five deglycosylated full-length IgG3 models. The corresponding five glycosylated IgG3 models were created by adding complex-type biantennary glycans to the two Asn-297 side chains in the Fc region (Fig. 1*B*). The five glycosylated IgG3 starting structures were each energy minimized.

**Figure 9:**
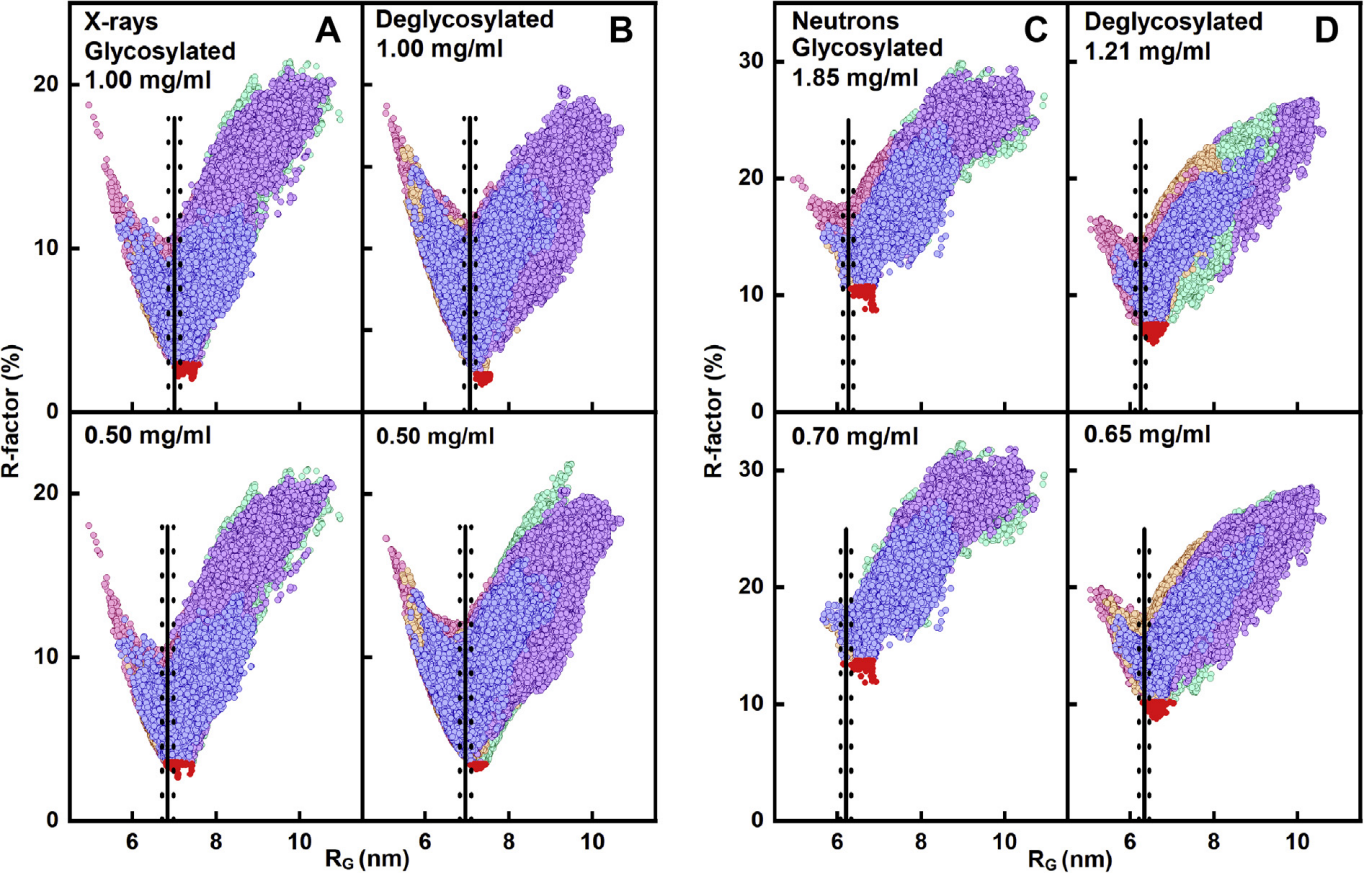
**Atomistic modeling analyses for glycosylated and deglycosylated IgG3.** The R-factor values for a total of 135,135 and 73,905 physically realistic models for glycosylated and deglycosylated IgG3 are shown as *circles* when these were plotted against the theoretical *R*_*G*_ values. The models were generated from five glycosylated start structures, which independently generated 34,315, 29,165, 27,635, 24,425, and 19,595 structures and these were then combined to give a total of 135,135 accepted structures. A total of 73,905 deglycosylated models were accepted from five structures that independently generated 13,345, 16,200, 7665, 21,375, and 15,320 structures. The five starting IgG3 models (Experimental procedures) from which the TAMC structures were derived have different hinge conformations. The five sets of structures are colored in *purple*, *green*, *orange*, *pink*, and *blue*. *A* and *B*, two experimental X-ray scattering curve analyses for two concentrations of glycosylated and deglycosylated IgG3 are shown. *C* and *D*, two experimental neutron scattering curve analyses for two concentrations of glycosylated and deglycosylated IgG3 are shown. In all eight panels, the experimental curves were fitted to the total of 135,135 and 73,905 modeled curves. Those models with *R*_*G*_ values closest to the experimental *R*_*G*_ values showed the lowest goodness-of-fit *R*-factor as expected. The 100 best-fit models with the lowest goodness-of-fit *R*-factors are denoted by *red circles*. The experimental *R*_*G*_ value is represented by the *solid vertical line*, and the *dashed vertical lines* on either side of this represent the ±2% upper and lower limits of these *R*_*G*_ values.

Physically realistic IgG3 models without steric overlaps or clashes were created for comparison with the experimental X-ray curves. By varying the torsion angles at three flexible regions at the start and end of the two IgG3 hinges (Experimental procedures) (Fig. 1), trial IgG3 structures were created that involved movements of the two Fab and one Fc regions relative to each other. For glycosylated IgG3, 250,000 models were generated in five Monte Carlo simulations, from which 135,135 models were accepted because these showed no steric clashes. For deglycosylated IgG3, 250,000 models were generated in five Monte Carlo simulations, of which 73,905 models were likewise acceptable. As a control to ensure that no systematic trends had been overlooked in the datasets, four X-ray and four neutron scattering curves for up to four concentrations were fitted for each of the four samples in question (Fig. 9).

(a) For X-rays with both forms of IgG3, comparison of the four experimental scattering curves at 0.50 to 1.00 mg/ml with the 135,135 and 73,905 theoretical curves gave a goodness-of-fit *R*-factor *versus R*_*G*_ distribution with clear minima in all eight cases (Fig. 9, *A* and *B*). The minima agreed with the experimental *R*_*G*_ values (Fig. 6*A*). The minima showed that enough trial X-ray models had been generated to result in good fits in each case. Filtering of the models to select these with the lowest *R*-factors gave the 100 best-fit models for each concentration (red, Fig. 9). The range of the 100 *R*-factors for each of the concentrations was low at between 2.04% and 3.58% for the best-fit glycosylated models and between 1.70% and 3.47% for the best-fit deglycosylated models (Table 2). This indicated good-quality X-ray curve fits between the experimental and modeled curves.
(b) For neutrons, the same outcome was found, thus confirming the reproducibility of the curve fits. Comparisons of the same 135,135 and 73,905 theoretical curves with the neutron scattering curves at 0.65 to 1.85 mg/ml showed again that 100 best-fit structures could be identified at clear minima in each of the *R*-factor *versus R*_*G*_ neutron distributions (Fig. 9, *C* and *D*). The minima agreed with the experimental *R*_*G*_ values (Fig. 6*B*). The range of R-factors was now higher than the X-ray values because of the reduced precision of the neutron scattering curves.

**Table 2:** Modeling fits for the X-ray scattering and analytical ultracentrifugation data in light water

Filter	Models	R_*G*_ before minimization (nm)	R_*G*_ after minimization (nm)	R_*XS-1*_ before minimization (nm)	R_*XS-1*_ after minimization (nm)	R_*XS-2*_ before minimization (nm)	R_*XS-2*_ after minimization (nm)	*L* (nm)	R-factor before minimization (%)	R-factor after minimization (%)	*s*_*20,w*_ before minimization (S)	*s*_*20,w*_ after minimization (S)
Library of glycosylated models	87,200	4.96–10.97	NA	NA	NA	NA	NA	NA	2.04–21.48	NA	NA	NA
Top 100 at 1.00 mg/ml	100	7.02–7.58	6.98–7.54	2.04–2.81	1.89–2.67	1.30–1.67	1.34–1.71	NA	2.04–2.93	2.09–4.44	6.03–6.51	6.03–6.51
Best fit at 1.00 mg/ml	1	7.43	7.38	2.24	2.10	1.45	1.45	28	2.04	2.80	6.30	6.30
Top 100 at 0.50 mg/ml	100	6.80–7.43	6.79–7.39	2.04–2.79	1.89–2.67	1.36–1.77	1.34–1.71	NA	2.66–3.58	2.62–4.84	6.07–6.61	6.07–6.61
Best fit at 0.50 mg/ml	1	7.09	7.06	2.26	2.19	1.57	1.55	25	2.66	2.89	6.49	6.49

Library of deglycosylated models	135,141	5.06–10.68	NA	NA	NA	NA	NA	NA	1.70–21.8	NA	NA	NA
Top 100 at 1.00 mg/ml	100	7.22–7.56	7.20–7.55	2.29–2.71	2.42	1.34–1.70	1.33–1.67	NA	1.70–2.32	1.77–3.20	5.69–5.99	5.74–6.00
Best fit at 1.00 mg/ml	1	7.36	7.35	2.26–2.69	2.39	1.33–1.67	1.59	27	1.70	1.89	5.92	5.94
Top 100 at 0.50 mg/ml	100	7.09–7.45	7.08–7.44	2.10–2.54	2.08–2.48	1.38–1.72	1.15–1.73	NA	3.17–3.47	3.12–4.52	5.75–6.06	5.87–6.07
Best fit at 0.50 mg/ml	1	7.23	7.22	2.27	2.25	1.55	1.55	27	3.17	3.19	6.00	6.00

PCA group 1	50	6.80–7.58	6.79–7.54	2.34–2.79	2.13–2.67	1.30–1.65	1.34–1.73	NA	2.68–3.58	2.45–4.84	6.18–6.52	6.18–6.52
Glycosylated	50	6.80–7.58	6.79–7.54	2.34–2.79	2.13–2.67	1.30–1.65	1.34–1.73	NA	2.68–3.58	2.45–4.84	6.18–6.52	6.18–6.52
Deglycosylated	0	NA	NA	NA	NA	NA	NA	NA	NA	NA	NA	NA
Centroid	1	6.99	6.9	2.77	2.49	1.49	1.51	21	3.53	3.34	6.37	6.37

PCA group 2	50	7.29–7.56	7.25–7.54	3.04–2.69	1.89–2.58	1.34–1.66	1.33–1.63	NA	1.95–3.48	1.88–4.44	5.69–6.36	5.74–6.36
Glycosylated	31	7.33–7.50	7.25–7.45	2.04–2.57	1.89–2.33	1.34–1.66	1.35–1.63	NA	2.04–3.48	2.23–4.44	6.15–6.36	6.15–6.36
Deglycosylated	19	7.29–7.56	7.27–7.54	2.24–2.69	2.19–2.58	1.37–1.53	1.33–1.52	NA	1.95–3.45	1.88–3.44	5.69–5.93	5.74–5.94
Centroid	1	7.33	7.26	2.57	2.32	1.56	1.49	22	2.85	3.15	6.24	6.24

PCA group 3	83	6.84–7.16	6.83–7.08	2.08–2.61	2.01–2.56	1.36–1.77	1.15–1.74	NA	2.20–3.58	2.46–4.52	5.85–6.61	5.89–6.61
Glycosylated	81	6.84–7.10	6.83–7.08	2.08–2.61	2.01–2.56	1.36–1.77	1.34–1.74	NA	2.20–3.58	2.46–4.10	6.32–6.61	6.32–6.61
Deglycosylated	2	7.16	7.08	2.52–2.54	2.40–2.42	1.44–1.46	1.15	NA	3.45–3.46	4.51–4.52	5.85–5.86	5.89–5.90
Centroid	1	6.88	6.87	2.11	2.05	1.53	1.48	24	3.54	4.06	6.60	6.60

PCA group 4	38	7.07–7.35	6.99–7.27	2.57–2.81	2.38–2.67	1.41–1.67	1.45–1.71	NA	2.29–3.56	2.09–3.57	6.03–6.27	6.03–6.27
Glycosylated	38	7.07–7.35	6.99–7.27	2.57–2.81	2.38–2.67	1.41–1.67	1.45–1.71	NA	2.29–3.56	2.09–3.57	6.03–6.27	6.03–6.27
Deglycosylated	0	NA	NA	NA	NA	NA	NA	NA	NA	NA	NA	NA
Centroid	1	7.24	7.13	2.81	2.6	1.53	1.55	23	2.93	2.36	6.19	6.19

PCA group 5	179	7.09–7.56	7.08–7.55	2.10–2.71	2.08–2.69	1.34–1.72	1.33–1.73	NA	1.70–3.47	1.78–3.70	5.71–6.06	5.78–6.07
Glycosylated	0	NA	NA	NA	NA	NA	NA	NA	NA	NA	NA	NA
Deglycosylated	179	7.09–7.56	7.08–7.55	2.10–2.71	2.08–2.69	1.34–1.72	1.33–1.73	NA	1.70–3.47	1.78–3.70	5.71–6.06	5.78–6.07
Centroid	1	7.40	7.39	2.59	2.58	1.5	1.48	23	2.23	2.06	5.83	5.83

Abbreviation: NA, not available.

The eight sets of 100 best-fit X-ray and neutron models (Fig. 9) were subjected to PCA in order to identify the resulting best-fit IgG3 conformations from the curve fits (26). The PCA determines the correlated motions of protein residues in IgG3 as linearly uncorrelated variables, each being termed a principal component (25). These “essential motions” were extracted from a covariance matrix of the atomic coordinates of the frames in the selected IgG3 structure set. The eigenvectors of this matrix each have an associated eigenvalue that characterizes the clustering of the models based on structural coordinates (or variance). In order to eliminate bias in the PCA, the glycan chains were removed from the glycosylated IgG3 models before comparison with the deglycosylated models.

**Figure 10:**
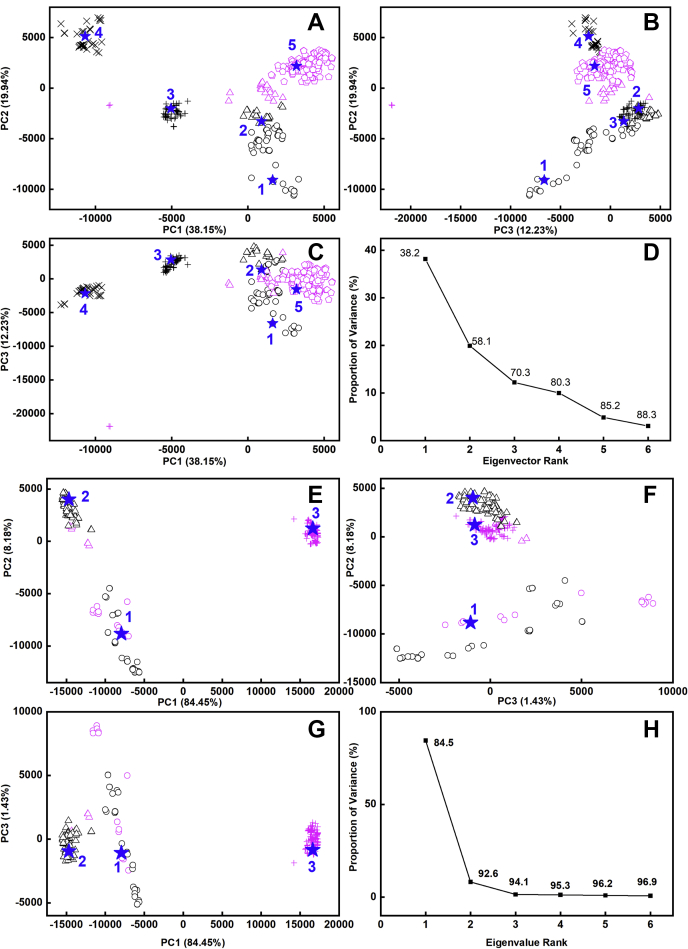
**Principal component analyses (PCA) of the best-fit glycosylated and deglycosylated IgG3 models.** Glycosylated models are represented by *black symbols*, and deglycosylated models are represented by *magenta symbols*. The PCA groups 1, 2, 3, 4, and 5 are represented by ○, Δ, +, ×, and ⌂ in that order, and the centroid model for each group is represented by *blue* numbered ★ symbols. *A–D*, the four sets of 100 best-fit models from the experimental X-ray scattering curves for glycosylated and deglycosylated IgG3 were grouped by PCA into four groups of PC2 *versus* PC1, PC3 *versus* PC2, and PC3 *versus* PC1. *D*, the first three eigenvalue rankings (PC1, PC2, and PC3) captured 38.2% of the variance in the 400 models. *E–H*, the four sets of 100 best-fit models from the experimental neutron scattering curves for glycosylated and deglycosylated IgG3 were grouped by PCA into four groups PC2 *versus* PC1, PC3 *versus* PC2, and PC3 *versus* PC1. *H*, The first three eigenvalue rankings (PC1, PC2, and PC3) captured 84.5% of the variance in the 400 models.

**Figure 11:**
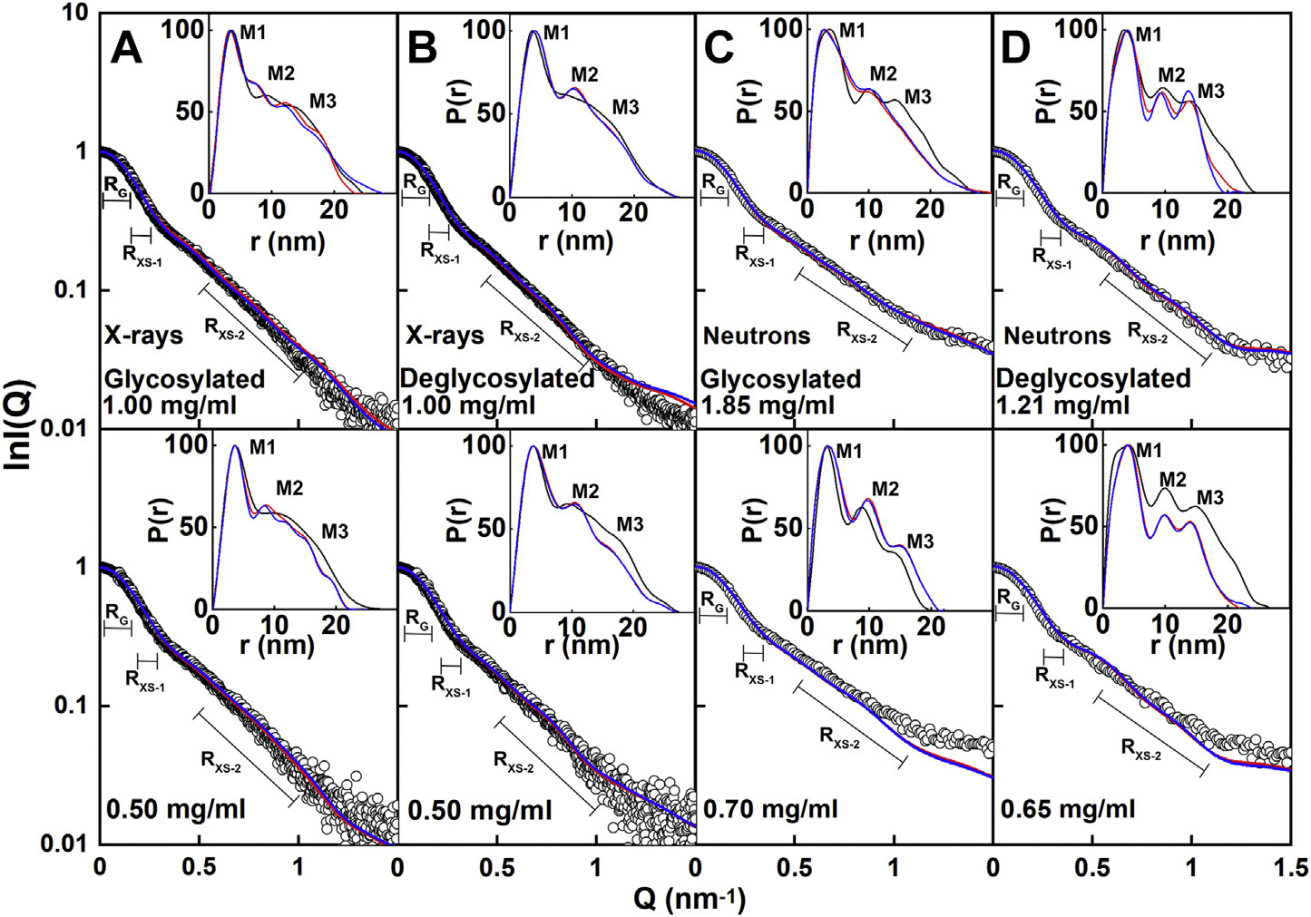
**Scattering curve fits for the best-fit glycosylated and deglycosylated IgG3 models.** The experimental curves are denoted by *black circles*. The Guinier *Q* ranges used in Figure 5 for the *R*_*G,*_*R*_*XS-1*_, and *R*_*XS-2*_ fit ranges are denoted by *black horizontal bars*. The best-fit modeled curves are denoted by *red lines*, and the best-fit modeled curves after energy minimization denoted by *blue lines*. The corresponding distance distribution curves *P(r)* are shown in the *top right* of each panel and labeled with the *M1*, *M2*, and *M3* peaks where visible. *A*, the glycosylated and (*B*) deglycosylated IgG3 X-ray curve fits are shown. In *A*, the glycosylated IgG3 model fits were taken from PCA group 2 (1.00 mg/ml) and PCA group 1 (0.50 mg/ml) (Table 2). In *B*, the deglycosylated IgG3 model fits were taken from PCA group 5 (1.00 and 0.50 mg/ml) in that order. The X-ray experimental curves corresponding to glycosylated IgG3 at 1.00 mg/ml and deglycosylated IgG3 at 1.00 mg/ml and their modeled curve fits are available in supporting information. *C*, the glycosylated and (*D*) deglycosylated IgG3 neutron curve fits are shown. A flat background correction of 2.0% of I(0) was applied to allow for incoherent scattering. In *C*, the glycosylated IgG3 model fits corresponded to PCA group 2 (1.85 and 0.70 mg/ml) (Table 3). In *D*, the deglycosylated IgG3 model fits corresponded to PCA group 3 (1.21 and 0.65 mg/ml).
(b) For the neutron fits, the PCA also indicated differences between the glycosylated and deglycosylated IgG3 models (black and magenta, respectively, Fig. 10, *E–H*; Table 3). The best-fit 200 glycosylated and 200 deglycosylated neutron models were clustered into three groups, with some overlap between glycosylated and deglycosylated groups. The glycosylated models were mostly in PCA groups 1 and 2, whereas the deglycosylated models were mostly in PCA group 3 but with some overlap with PCA group 1. Visually good neutron *I(Q)* curve fits were obtained; however, the fit of these curves was inferior to the fit obtained in the X-ray fits at high *Q* (Fig. 11, *C* and *D*). An incoherent scattering flat background correction of 2.0% of *I(0)* in the neutron fits accounted for a mismatch between the sample and the buffer background in heavy water (27). Of interest, in all four neutron fits, the triple-peaked *P(r)* curves were well replicated in the modeled best-fit curves.

(a) For the X-ray fits, the PCA indicated a difference between the glycosylated and deglycosylated IgG3 models (black and magenta, respectively, Fig. 10, *A–D*; Table 2). Thus, the best-fit 200 glycosylated and 200 deglycosylated X-ray models were each clustered into five distinct groups, The glycosylated models mostly occurred in the PCA groups 1, 2, 3, and 4, whereas the deglycosylated models mostly occurred in the PCA group 5 with some overlap with group 2. The visually excellent ln *I(Q)* and *P(r)* X-ray curve fits confirmed the validity of the modeling fits (Fig. 11, *A* and *B*).

**Table 3:** Modeling fits for the neutron scattering and analytical ultracentrifugation data in light water

Filter	Models	R_*G*_ before minimization (nm)	R_*G*_ after minimization (nm)	R_*XS-1*_ before minimization (nm)	R_*XS-1*_ after minimization (nm)	R_*XS-2*_ before minimization (nm)	R_*XS-2*_ after minimization (nm)	*L* (nm)	R-factor before minimization (%)	R-factor after minimization (%)	*s*_*20,w*_ before minimization (S)	s_20,w_ after minimization (S)
Library of glycosylated models	87,200	4.95–10.94	NA	NA	NA	NA	NA	NA	8.72–32.29	NA	NA	NA
Top 100 at 1.85 mg/ml	100	6.33–6.93	6.32–6.89	1.73–2.37	1.70–2.34	1.25–2.09	1.28–2.09	NA	8.72–10.71	8.07–10.73	6.32–6.75	6.51–6.74
Best fit at 1.85 mg/ml	1	6.93	6.89	1.93	1.84	1.30	1.33	23	8.72	8.07	6.62	6.73
Top 100 at 0.70 mg/ml	100	6.14–6.93	6.13–6.89	1.73–2.29	1.69–2.29	1.25–2.09	1.28–2.09	NA	11.82–13.73	11.30–13.66	6.32–6.75	6.51–6.75
Best fit at 0.70 mg/ml	1	6.19	6.65	1.84	1.82	1.49	1.51	25	11.82	11.77	6.64	6.7

Library of deglycosylated models	135,141	5.04–10.62	NA	NA	NA	NA	NA	NA	5.90–28.52	NA	NA	NA
Top 100 at 1.21 mg/ml	100	6.30–6.86	6.27–6.81	1.79–2.62	1.76–2.59	1.21–1.96	1.21–1.97	NA	5.90–7.51	5.56–7.35	6.02–6.41	6.11–6.42
Best fit at 1.21 mg/ml	1	6.56	6.62	2.35	2.32	1.58	1.57	22	5.90	6.76	6.15	6.16
Top 100 at 0.65 mg/ml	100	6.30–7.04	6.27–7.02	1.79–2.62	1.76–2.59	1.21–1.95	1.20–1.96	NA	8.75–10.17	8.57–10.10	5.95–6.40	6.00–6.42
Best fit at 0.65 mg/ml	1	6.62	6.6	2.1	2.06	1.67	1.67	21	8.75	8.57	6.31	6.32

PCA group 1	68	6.32–7.04	6.32–7.02	1.83–2.37	1.79–2.36	1.50–2.09	1.50–2.36	NA	6.66–13.70	6.41–13.66	5.95–6.75	6.00–6.74
Glycosylated	49	6.33–6.76	6.32–6.76	1.92–2.37	1.93–2.34	1.54–2.09	1.55–2.09	NA	9.90–13.70	9.40–13.66	6.32–6.75	6.51–6.74
Deglycosylated	19	6.32–7.04	6.32–7.02	1.83–2.36	1.79–2.36	1.50–1.85	1.50–2.36	NA	6.66–10.16	6.41–10.09	5.95–6.34	6.00–6.34
Centroid	1	6.63	6.63	2.04	2.00	1.79	1.80	21	7.39	6.93	6.25	6.32

PCA group 2	159	6.14–6.93	6.13–6.89	1.73–2.29	1.69–2.19	1.25–1.74	1.22–2.15	NA	7.08–13.73	6.23–13.64	6.06–6.70	6.17–6.75
Glycosylated	151	6.14–6.93	6.13–6.89	1.73–2.13	1.69–2.07	1.25–1.74	1.28–1.74	NA	8.72–13.73	8.07–13.64	6.50–6.70	6.56–6.75
Deglycosylated	8	6.40–6.81	6.37–6.79	1.79–2.29	1.76–2.19	1.28–1.50	1.22–2.15	NA	7.08–10.15	6.23–9.63	6.06–6.28	6.17–6.36
Centroid	1	6.66	6.64	1.91	1.89	1.32	1.31	22	1.37	1.35	6.59	6.65

PCA group 3	173	6.30–6.79	6.27–6.77	1.90–2.62	1.85–2.59	1.21–1.96	1.21–2.59	NA	5.91–10.17	5.56–10.10	6.10–6.41	6.11–6.42
Glycosylated	0	NA	NA	NA	NA	NA	NA	NA	NA	NA	NA	NA
Deglycosylated	173	6.30–6.79	6.27–6.77	1.90–2.62	1.85–2.59	1.21–1.96	1.21–2.59	NA	5.91–10.17	5.56–10.10	6.10–6.41	6.11–6.42
Centroid	1	6.74	6.66	1.93	1.88	1.86	1.83	21	7.30	6.41	6.31	6.32

Abbreviation: NA, not available.

**Figure 12:**
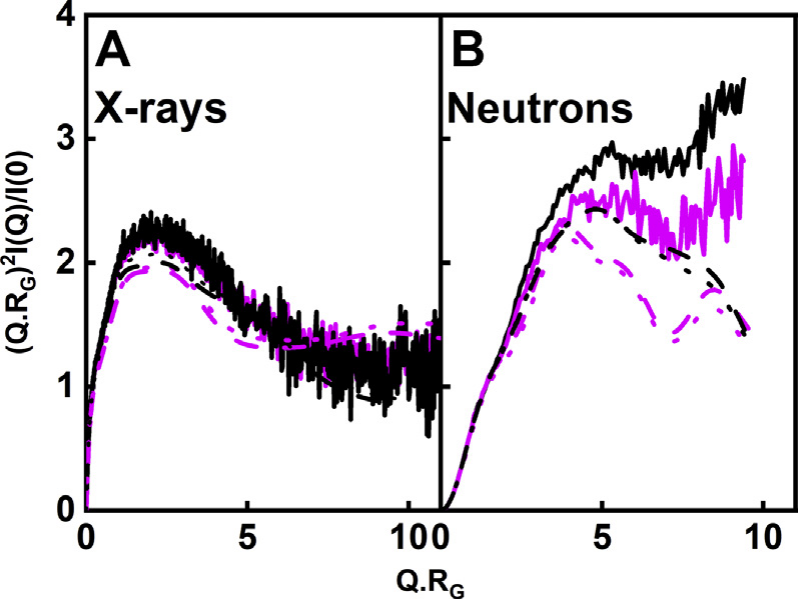
**Normalized Kratky plots for the experimental and best-fit glycosylated and deglycosylated IgG3 scattering curves.***A*, X-ray experimental data (*solid lines*) and model fits before energy minimization (*dashed lines*) and model fits after energy minimization (*dotted lines*) were shown in black for glycosylated IgG3 at 1.00 mg/ml and in magenta for deglycosylated IgG3 at 1.00 mg/ml. *B*, neutron experimental data (*solid lines*) and model fits before energy minimization (*dashed lines*) and model fits after energy minimization (*dotted lines*) were shown in *black* for glycosylated IgG3 at 1.85 mg/ml and in magenta for deglycosylated IgG3 at 1.21 mg/ml.

Further insights into the X-ray and neutron data and their modeling were obtained from the dimensionless Kratky analyses of *(Q·R*_*G*_*)*^*2*^*·I(Q)/I(0) versus Q·R*_*G*_ for the experimental scattering curves at the highest concentrations in use and the scattering curves from the modeled best fit structures. These plots indicate whether the macromolecule in question is globular in its structure or possesses intrinsically disordered regions (28). Unlike monoclonal IgG1 and myeloma IgG2 where two clear Kratky peaks were observed at around 2 and 4 nm (16, 17), only a single Kratky peak close to 2 nm was observed for myeloma IgG3. For the X-ray Kratky curves (Fig. 12*A*), the *Q·R*_*G*_ values for the experimental glycosylated IgG3 peak was 2.24, in good accord with the modeled values of 2.25 before energy minimization and 2.23 after energy minimization. The *Q·R*_*G*_ values for the experimental deglycosylated peaks of 2.26 were also in good accord with the modeled deglycosylated peaks of 2.26 before energy minimization and 2.33 after energy minimization. For the neutron Kratky curves (Fig. 12*B*), the *Q·R*_*G*_ value for the experimental glycosylated IgG3 peak was shifted to 4.79, which were similar to the modeled peaks at 4.80 before energy minimization and 4.80 after energy minimization. For deglycosylated IgG3, the *Q·R*_*G*_ value for the experimental peak was 4.66, which was larger than the modeled values of 3.88 before and after energy minimization. The existence of a single and not double Kratky peak for IgG3 was most likely due to the large separation between the Fab and Fc regions in IgG3, meaning that these three regions showed independent scattering properties (7).

As another test of the scattering modeling, the *s*^*0*^_*20,w*_ values for the four sets of best-fit 100 glycosylated and deglycosylated models from each X-ray concentration (Figs. 9 and 11) were calculated using HullRad (29). This gave an *s*^*0*^_*20,w*_ range of 6.03 to 6.61 S for the four X-ray concentrations for glycosylated IgG3 and 5.74 to 6.07 S for deglycosylated IgG3 (Table 2). These values agreed well with the experimental *s*^*0*^_*20,w*_ values of 5.90 to 5.78 S for glycosylated IgG3 and 6.24 to 6.33 S for deglycosylated IgG3 (Table 1). These agreements corroborated the outcome of the atomistic scattering modeling, given that the mean difference between the modeled and experimental values should typically be ±0.21 S for related macromolecules (30). This modeling was, however, unable to distinguish changes before and after deglycosylation.

## Discussion

**Figure 13:**
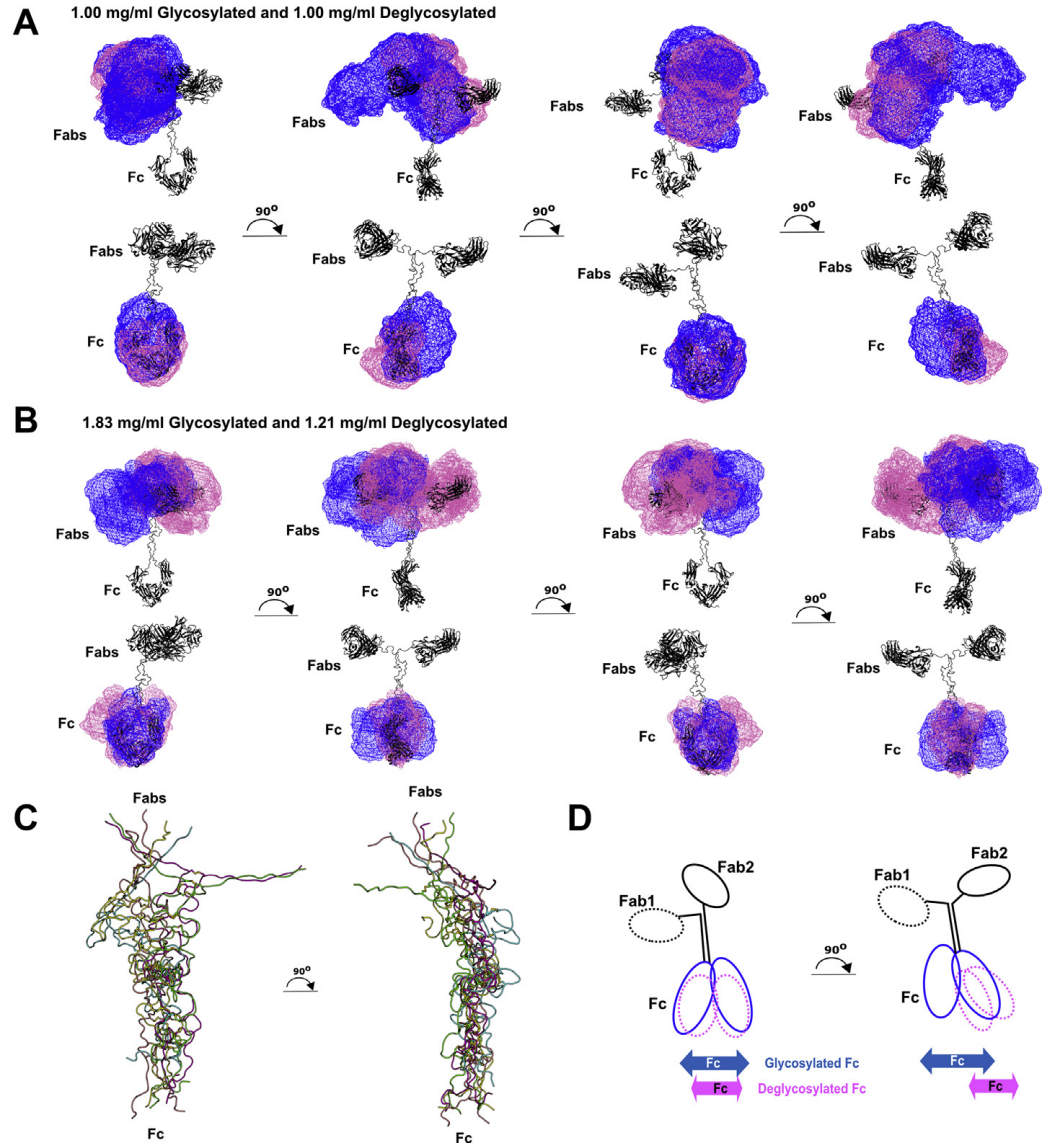
**Views of representative best-fit X-ray and neutron structures.** The *black cartoon* denotes the protein backbone of the starting glycosylated and deglycosylated IgG3 models. In the *upper row* of *A* and *B*, the Fc regions of the 100 best-fit models were superimposed onto the starting reference structure, thus focusing on variations in the Fab region in these models. In the *lower row* of *A* and *B*, the Fab regions of the 100 best-fit models were superimposed onto this starting reference structure, thus focusing on variations in the Fc region in these models. The *blue* and *magenta* wireframe envelopes denote the conformational space occupied by the glycosylated and deglycosylated Fab or Fc regions. *A*, Best-fit X-ray models at 1.00 mg/ml for both IgG3 forms. *B*, Best-fit neutron models at 1.83 and 1.23 mg/ml IgG3. *C*, superimposition of the five hinge structures used for the IgG3 modeling. *D*, cartoon to illustrate the best-fit IgG3 structure from this study, adapted from (*A*).

The first experimentally determined molecular structure for a full-length IgG3 antibody was determined by this joint AUC, SAXS, and SANS study of human myeloma IgG3, combined with an atomistic scattering curve fit method based on molecular dynamics and Monte Carlo simulations (27). Its solution structure was shown to possess a semirigid hinge with little disorder that resulted in a wide separation between the Fab and Fc regions in IgG3. This study now completes a series of similar studies on all four human IgG subclasses IgG1 to IgG4 that resulted in the first atomistic experimentally determined solution structures for all of these and clarified their molecular functions (16, 17, 26). Unlike previous studies of the full-length IgG3 structure, our approach utilized simulations of stereochemically correct atomistic models using a combination of molecular dynamics and Monte Carlo simulations to fit the experimental data. The simulations resulted in conformational libraries of 135,135, and 73,905 models that explored a wide range of structures for full-length IgG3. When these libraries were compared with extensive experimental datasets from SAXS and SANS, it was possible to rank these models in terms of goodness of fit to the experimental scattering curves. In order to show the relative positions of the Fab and Fc regions in the top best-fit 100 models, these structures were superimposed upon each other at their hinge, then were displayed as wireframe representations rotated about their vertical axis (Fig. 13). These views make it clear that the IgG3 solution structure involves an extended but not rigid polyproline hinge with some flexibility in this and that the two Fab regions and the one Fc region each occupy distinct conformational space about the hinge. Unlike glycosylated and deglycosylated IgG1 (17), no clear differences between these two forms in IgG3 were detectable. This outcome is most likely attributable to the length of the hinge region, which may reduce the impact of any conformational differences in flexibility that was previously seen for IgG1 (17).

The most prominent feature of the IgG3 models is the polyproline disulfide-linked hinge region (Fig. 1*A*). For the curve fits, five such hinges were simulated using energy minimization and molecular dynamics, and all showed extended but variable structures (Fig. 13*C*). The maximum length of the hinge between Glu216 and Pro285 (Fig. 2*E*) ranged between 7.67 and 10.80 nm, in good agreement with the increased length of IgG3 compared with IgG1, IgG2, and IgG4. The simulations do not predict a regular polyproline helix structure with no flexibility, and this outcome concurs with the scattering curves.

Experimentally, the elongated semirigid IgG3 solution structure was primarily confirmed from the Guinier scattering data, where the *R*_*G*_ values derived from Guinier analyses were significantly larger at 6.97 nm by X-rays and 6.26 nm (Table 1) than the *R*_*G*_ values reported for the other IgG subclasses, namely, IgG1 at 5.02 to 5.32 nm, IgG2 at 5.24 to 5.38 nm, and IgG4 at 4.77 to 4.94 nm (8, 16, 17, 22, 23) (Fig. 6). The cross-sectional radius, *R*_*XS-1*_, that typically monitored the spatial separation of the Fab and Fc regions in IgG was smaller at 1.41 nm by X-ray for IgG3 compared with those of the other IgG subclasses. Thus the other X-ray *R*_*XS-1*_ values were 2.46 to 2.65 nm for IgG1 (17, 23), 2.61 nm for IgG2 (16), and 2.30 to 2.50 nm for IgG4 (22). The significant decrease in the *R*_*XS-1*_ value for IgG3 compared with IgG1, IgG2, and IgG4 is attributable to the separation of the Fab and Fc regions by the elongated hinge of IgG3. The *R*_*XS-2*_ parameter represents the averaged spatial cross-section of each of the two Fab and one Fc regions in IgG1, IgG2, and IgG4. This was 1.61 nm in IgG3, which is similar to those of the other IgG subclasses, namely, 1.35 to 1.43 nm for IgG1, 1.35 to 1.37 nm for IgG2, and 1.23 to 1.42 nm for IgG4. In fact, the *R*_*XS-1*_ value of IgG3 is similar to the *R*_*XS-2*_ values of all four IgG subclasses, suggesting that this parameter may correspond to the apparent cross-section of the two Fab regions positioned in a linear elongated arrangement shown in Figure 13, *A* and *B*.

Further evidence for the formation of an elongated IgG3 solution structure compared with IgG1, IgG2, and IgG4 was obtained from the distance distribution function *P(r)*. The maximum length *L* of IgG3 was significantly larger at 25 to 28 nm (Fig. 7) than the *L* values for the other IgG subclasses, IgG1, namely, 16 to 17 nm for IgG1, 17 nm for IgG2, and 15 to 16 nm for IgG4. The extra length *L* for IgG3 suggested that the IgG3 hinge is about 10 nm long. In addition, the appearance of the *P(r)* curve for IgG3 showed three maxima *M1*, *M2*, and *M3*, whereas the other IgG subclasses only showed two maxima *M1* and *M2*. The three maxima in the *P(r)* curves for IgG3 were well replicated by the atomistic modeling (Fig. 11). In molecular terms, the *M1* maximum is attributed to the distances within a single Fab or Fc region, whereas the *M2* and *M3* maxima are attributed to the separation between the two Fab regions and the separations of the Fab-Fc regions, respectively. *M2* and *M3* are thus conformational monitors of the overall extended IgG3 structure, which is relatively well defined in its appearance despite some conformational variability seen in its hinge. The different *M3* values seen by X-ray and neutrons suggested that, whereas the IgG3 hinge can adopt a range of conformations, the IgG3 hinge remained extended in solution. Kratky plots are often used as a measure of structural flexibility (28). For IgG3, only one peak was now visible (Fig. 12), instead of the two peaks seen for IgG1, IgG2, and IgG4, and no effect was seen following glycan removal. The best-fit theoretical models showed good agreement with the experimental SAXS curve, again indicating that the best-fit atomistic modeling was able to replicate the Kratky peak. No increase in flexibility was detected after glycan removal, unlike IgG1 and IgG4. A previous study analyzed X-ray Kratky plots for IgG1, IgG2, and IgG3 to propose that IgG3 has increased flexibility when compared with IgG1 and IgG2 (7). Our analyses do not support this extra flexibility because (i) we are able to observe a well-defined *M3* peak by X-rays and neutrons, which would not be possible if the structure was very flexible (Fig. 7), and (ii) good curve fits for the *I(Q)*, *P(r)*, and Kratky curves were obtained with a conformationally variable but essentially static set of IgG3 molecular models (Figs. 11 and 12).

The experimental AUC data for IgG3 provided another clear indication that its solution structure is more elongated than those of IgG1, IgG2, and IgG4. Data from AUC, SAXS, and SANS provided excellent insight into the glycosylated and the deglycosylated structure of human IgG3. The AUC data showed that IgG3 was monomeric in solution up to 2 mg/ml. The lower sedimentation coefficients *s*_*20,w*_ of 6.0 S compared with IgG1 (6.5 S), IgG2 (7.2 S), and IgG4 (6.5 S) demonstrated that IgG3 has a more elongated structure than IgG1, IgG2, and IgG4 (17, 26).

The present application of atomistic scattering modeling resulted in molecular structures that were able to reproduce all the major features of the SAXS, SANS, and AUC data collection. The datasets were fitted to a library of 135,135 and 73,905 trial models. The best-fit X-ray R-factors were low at 2.04% and 1.70% (Table 2). This R-factor improvement is attributed to the improved signal–noise ratio of the scattering curves from the B21 instrument at Diamond. The lowest R-factors for the neutron fits were higher because of the weaker signal–noise ratios, these being 8.72% and 5.90% (Table 3). One noteworthy aspect of the modeling study using SasCalc (31) is that an atomistic representation of hydration shells was not made because this was computationally expensive. SAXS sees these hydration shells as they have higher scattering densities similar to those of the protein, compared with bulk water (18, 19, 20). The hydration shell is less visible by SANS because its neutron scattering density is reduced and is similar to that of the heavy water buffer in use. The complementary fits of the SAXS and SANS datasets are thus a control that similar IgG3 models fit the scattering curves. We note that our previous use of the SCT/SCTPL modeling approach (available in SASSIE-web) for X-ray and neutron fits explicitly incorporated hydration shells in a coarse-grained approach.

IgG3 is a potent antibody with a high affinity for several FcRs and C1q. Its potency may explain the reduced concentration of IgG3 in plasma, compared with IgG1 and IgG2. The absence of the Fc glycan increased the conformational space occupied by the Fc region in studies of deglycosylated IgG1 (17). The IgG1 study concluded that the Asn297 glycans limit the conformational flexibility of the Fc region, stabilizing it in a conformation that makes receptor binding more likely (8). In this IgG3 study, conformational change in the Fc region was not detectable after deglycosylation (Fig. 13*D*). The longer hinge may enable the Fc glycans to play a more local role in stabilizing the C_H_2 domain pair, and we note the loss of FcγR receptor binding in aglycosylated IgG3 (15). The elongated hinge may stabilize the IgG3 Fc region by allowing any Fab motions to take place independently of the Fc region, which is not possible in the other IgG subclasses as they have shorter hinges. If the Fab and Fc regions are more independent of each other because of a longer hinge, this would explain the potency of IgG3, *i.e.*, the increased FcγR receptor and complement C1q affinity to IgG3, and why IgG4 with the shortest hinge in the IgG subclasses has the lowest affinity to the FcγR receptors.

More detailed comparisons of our IgG3 results with the recent literature are summarized:

(i) Recently it has been reported that human IgG1 associates at cell surfaces to form Fc hexamers, with one Fab region bound to the antigenic surface and the other Fab region being unbound and located at the height of the Fc regions; this hexameric arrangement confers superior C1q complement activation (32, 33, 34). In distinction another study found that C1q binding is better activated with human IgG1 through bivalent Fab–antigen binding (35). In this context, the presence of IgG3 with its longer hinge and with similar antigen binding specificity in serum at 5% to 8% abundance will complicate the formation of these Fc hexamers on cell surfaces, as hexamers are unlikely to be formed by mixtures of IgG1 and IgG3 subclasses. This suggests a distinct role for IgG3 in terms of its affinity for the FcγR receptor and complement C1q, perhaps being able to bind bivalently to antigenic surfaces as the result of flexibility in its hinge region, and then being able to binding to FcγR and C1q.
(ii) The bivalent binding of antibody to precise patterns of hapten-coated antigen surfaces was studied for the four human IgG subclasses (36). Studies of a surface where two NIP hapten molecules were arranged at separations from 3 to 44 nm showed that the strongest bivalent binding for IgG3 was seen at 14- to 16-nm distances. Measurement of the separation of the tips of the Fab regions in our best-fit 800 models for IgG3 showed that these separations corresponded to a mean of 8 ± 2 nm in a full range of 2 to 19 nm (Gly54 in the V_H_ domains; Fig. 2*C*). This outcome indicated that our best fit IgG3 solution structures showed a similar range of Fab separations to those seen for these antigenic separations on a surface.
(iii) IgG3 has a distinct role in some human autoantibody-mediated diseases, *e.g.*, targeting structures around the Node of Ranvier in the central nervous system, where these autoantibodies are of the IgG3 isotype. In acute autoimmune neuropathy, IgG3 was detected as the predominant autoantibody in patients with Guillain–Barré syndrome (37). This may relate to a subclass switch from IgG3 to the other three IgG subclasses, in the course of which the disease becomes more chronic, and we speculate that this might be related to the long hinge of IgG3. Serum IgG3 was also found to be significantly increased in primary biliary cirrhosis, this liver disease being another autoimmune disorder (38, 39, 40).

## Experimental Procedures

### Purification and composition of IgG3

IgG3 κ HP3 (164.8 kDa) was prepared from human myeloma serum stored at −20 °C. The serum was thawed at 37 °C, and spun at 3000 rpm for 15 min A volume of 12.5 ml of clear serum was dialyzed into 1 L of 0.01 M phosphate buffer at pH 7.5, repeated three times. IgG was isolated from other serum components using a DE 52 Whatman (DEAE) column (12 cm × 12 cm), in 0.01 M phosphate buffer pH 7.7, varying the salt concentration sequentially (0.2, 0.3, 0.4, 0.05, and 0.06 M). The most IgG3 was found from the salt concentration eluate of 0.01 M. IgG3 was further purified using a 1 ml prepacked Staphylococcus Protein A coupled to a Sepharose column (Cytiva). Staphylococcus Protein A binds IgG1, IgG2 and IgG4, but not IgG3, which flowed through the column. The purity of IgG3 following purification from serum was confirmed using SDS-PAGE.

To remove the N-glycans from IgG3, enzymatic deglycosylation was achieved using peptide:N-glycosidase F (PNGase F) (35.5 kDa, New England Biolabs), selected owing to its ability to remove glycans completely from the glycosylated Asn residues (41). Thus 20 μl PNGase F (1850 activity units) was used in excess to deglycosylate 1000 μl of IgG3 (8.8 mg/ml) by incubation at 37 °C for 10 h. Each deglycosylated IgG3 sample was concentrated using Amicon Ultra-0.5 ml centrifugal filters (50 kDa cutoff), which simultaneously allowed the PNGase F to pass through the membrane. Immediately before AUC, SAXS, and SANS data collection and measurements, glycosylated and deglycosylated IgG3 were further purified by gel filtration to remove any nonspecific aggregates using a Super 6 Increase 10/300 GL column (Cytiva), then concentrated using Amicon Ultra-15 spin concentrators (50 kDa cutoff) and dialyzed at 4 °C into 20 mM L-histidine, 138 mM NaCl, and 2.6 mM KCl buffer, pH 6.0. This histidine buffer improved the stability of IgG3 in solution.

As no sequences were available for myeloma IgG3, a hypothetical sequence was created based on the sequence of the IgG4 A33 Fab regions (42) and the Fc sequence from the IgG3 Fc crystal structure (PDB ID: 5W38) (6). The hinge sequence was assumed to be ELKTPLGDTTHTCPRCP(EPKSCDTPPPCPRCP)_3_APELLGGP. The assumed IgG3 Fab sequence was aligned with the IgG4 b72.3 crystal structure (PDB ID: 1BBJ) (43) in order to build an IgG3 A33 Fab structure (Fig. 2). This multiple sequence alignment was generated using Clustal Omega software (44). The N-linked glycan at Asn297 on the C_H_2 domains were approximated as complex-type biantennary oligosaccharides with a Man_3_GlcNAc_2_ core and two NeuNAc.Gal.GlcNAc antennae (12). From this sequence, the molecular mass of glycosylated IgG3 was calculated to be 158.1 kDa, its unhydrated volume was 203.7 nm^3^, its hydrated volume was 245.9 nm^3^, its partial specific volume $\;\bar{v}$ was 0.776 ml/g, and its absorption coefficient was 12.4. The molecular mass of deglycosylated IgG3 was calculated to be 154.0 kDa, its unhydrated volume was 199.4 nm^3^, its hydrated volume was 241.5 nm^3^, its $\bar{v}$ was 0.780 ml/g, and its absorption coefficient was 12.8 (18). The X-ray and neutron scattering densities of glycan residues are similar to those for hydrophilic (polar) amino acid residues, these being slightly higher than those for hydrophobic (nonpolar) amino acid residues (18). The buffer density was measured on an Anton Paar DMA 5000 density meter at 20 °C to be 1.00578 g/ml in light water and 1.11106 g/ml in heavy water. Buffer viscosities were measured on an Anton Paar AMVn Automated microviscometer at 20 °C to be 0.010190 and 0.01384 P, respectively, for light and heavy water, pH 6.0.

The completeness of deglycosylation was verified by Superose 6 gel filtration, SDS-PAGE, and mass spectrometry. In the Mass Spectrometry Facility at the Chemistry Department University College London, the antibodies were analyzed on an Agilent 6510 Quadrupole time-of-flight liquid chromatography mass spectrometry system (Agilent). A volume of 10 μL of each sample was injected onto a PLRP-S, 1000A, 8 μM, 150 mm × 2.1 mm column, which was maintained at 60 °C at a flow of 0.3 ml/min. The separation was achieved using mobile phases A (water with 0.1% formic acid) and B (acetonitrile, with 0.1% formic acid) using a gradient elution. The column effluent was continuously electrosprayed into the capillary electrospray ionization source of the Agilent 6510 QTOF mass spectrometer, and electrospray ionization mass spectra were acquired in positive electrospray ionization mode using the *m/z* range 1000 to 3200 in profile mode. The raw data were converted to zero charge mass spectra using the maximum entropy deconvolution algorithm in the MassHunter software version B.07.00 (Agilent). The single glycan mass was found by subtracting the mass of fully glycosylated IgG3 from that for deglycosylated IgG3 and halving this mass.

### Sedimentation velocity data and analysis of IgG3

Analytical ultracentrifugation data for glycosylated and deglycosylated IgG3 were obtained on two Beckman XL-I instruments equipped with AnTi50 rotors. Data were collected at 20 °C, at a rotor speed of 30,000 rpm in two-sector cells with column heights of 12 mm for approximately 6 h. Sedimentation analyses were performed using direct boundary Lamm fits of up to 600 scans using SEDFIT (version 15.01b) (45, 46). SEDFIT resulted in size-distribution analyses *c(s)*, for which the algorithm assumes that all species have the same frictional ratio *f/f0*. The final SEDFIT analyses used a fixed resolution of 200 and optimized the *c(s)* fits by floating *f/f0* and the baseline until the overall root mean square deviations and visual appearance of the fits were satisfactory. The percentage of oligomers in the total loading concentration was derived using the *c(s)* integration function. The buffer viscosity and density were the experimental values. The observed *s* values were corrected to *s*_*20,w*_ by:

$$s_{20,w}=s_{T,B}\left(\frac{\eta_{T,B}}{\eta_{20,w}}\right)\frac{{\left(1-\;\bar{v}\rho \right)}_{20,w}}{{\left(1-\;\bar{v}\rho \right)}_{T,B}}$$

where *s* is the sedimentation coefficient, the subscripts _*T,B*_ refer to the temperature of the buffer and _*20,w*_ refers to water at 20 °C, *ρ* is the solvent density, *η* is the solvent viscosity, and $\;\bar{v}$ is as above.

### X-ray and neutron scattering data and analyses for IgG3

X-ray scattering data were obtained during one beam session (July 2019) on Instrument B21 at the Diamond Light Source at the Rutherford Appleton Laboratory, operating with a ring energy of 3 Gev and a beamline operational energy of 12.4 keV (47). A PILATUS 2M detector with a resolution of 1475 × 1679 pixels (pixel size of 172 × 172 μm) was used with a sample-to-detector distance of 4.01 m giving a *Q* range from 0.04 to 4 nm^−1^ (where *Q* = 4 π sin θ/λ; 2θ = scattering angle; λ = wavelength). The glycosylated (0.50–2.46 mg/ml) and deglycosylated (0.50–1.00 mg/ml) IgG3 samples in light water buffer were loaded onto a 96-well plate and placed into an EMBL Arinax sample holder (48, 49). This measurement condition showed the antibody molecule as a hydrated structure in a high positive solute–solvent contrast (18). An automatic sampler injected 30 μl of sample from the plate into a temperature-controlled quartz cell capillary with a diameter of 1.5 mm. Datasets of 30 frames with a frame exposure time of 1 s each were acquired in duplicate as a control of reproducibility. Checks during data acquisition confirmed the absence of radiation damage. An inhouse B21 pipeline termed DAWN (50) was used for buffer subtraction and data reduction, in which the 30 frames were averaged.

Neutron scattering data on glycosylated (0.70–1.85 mg/ml) and deglycosylated (0.65–1.21 mg/ml) IgG3 samples in heavy water buffer were obtained in one session (October 2020) on instrument SANS2D at the ISIS pulsed neutron source at the Rutherford Appleton Laboratory (51). This condition showed the antibody structure with a near-invisible hydration shell in a high negative solute–solvent contrast (18). No conformational differences in the antibody between light and heavy water were detected in this study or previously (52). A pulsed neutron beam was derived from proton beam currents of ∼40 μA. SANS2D data were recorded with 4 m of collimation, a 4-m sample-to-detector distance, a 12-mm sample aperture, and a wavelength range of 0.175 to 1.65 nm made available by a time of flight. This gave a *Q* range from 0.05 to 4 nm^−1^. The data were acquired using a two-dimensional ^3^He detector with 512 × 512 pixels of 7.5 × 7.5 mm^2^ size. Samples of volume 1 ml were measured in 2-mm path length circular banjo cells for 1 to 7 h in a thermostatted sample rack at 20 °C. Data were reduced using MANTID software (53). The MANTID data reduction steps include corrections for the *Q* resolution, *i.e.*, beam divergence effects and smearing from the shape and size of the slits, as well as the wavelength overlap in each pulse (53). Using SASview software, the Guinier analyses (below) were found to be almost unaffected if the smearing was turned on or off.

Guinier analyses of the scattering data give information of the radius of gyration *R*_*G*,_ the cross-sectional radius of gyration *R*_*XS*_, and the molecular mass. The scattering curve *I(Q)* intensities at low *Q* are defined by the *R*_*G*_ value, which is the averaged distance of each scattering point from the center of scattering. In a given solute–solvent contrast, the radius of gyration *R*_*G*_ is a measure of structural elongation if the internal inhomogeneity of scattering densities within the protein has no effect. Guinier analyses at low *Q* gave the *R*_*G*_ value and the forward scattering at zero angle *I(0)* (54):

$$\ln\;I\left(Q\right)=\ln\;I\left(0\right)-\frac{{R_{G}}^{2}Q^{2}}{3}$$

For antibodies, this expression is valid in a *Q·R*_*G*_ range up to 1.5 and was used in our previous studies (22, 23, 52), although the usual upper range reported in the literature is 1.0 to 1.3. If the structure is elongated, the mean radius of gyration of the cross-sectional structure *R*_*XS*_ and the mean cross-sectional intensity at zero angle [I (Q)Q]_Q_→_0_ is obtained from (54):

$$\ln\left[I\left(Q\right)Q\right]={\left[I\left(Q\right)Q\right]}_{Q\to 0}-\frac{{R_{XS}}^{2}Q^{2}}{2}$$

For the immunoglobulins IgG1, IgG2, and IgG4, it has been long recognized that the cross-sectional plot exhibits two regions, a steeper innermost one and a flatter outermost one (24), and the two analyses are denoted by *R*_*XS-1*_ and *R*_*XS-2*_, respectively. The *R*_*XS-1*_ parameter represents the averaged overall spatial separation of the Fab and Fc regions, this being one shorter Fab-Fab separation and two longer Fab–Fc separations (Fig. 1*A*), whereas the *R*_*XS-2*_ parameter represents the averaged spatial cross-section of each of the two Fab and one Fc regions. The *R*_*G*_ and *R*_*XS*_ analyses were performed using SCT (Table 1) (55). The *Q* ranges for the *R*_*G*_, *R*_*XS-1*_, and *R*_*XS-2*_ values were 0.10 to 0.22, 0.22 to 0.28, and 0.50 to 1.10 nm^−1^, respectively, these being distinct from IgG1, IgG2, and IgG4 (16, 17, 22, 23, 52). Indirect transformation of the scattering data *I(Q)* in reciprocal space into real space to give the distance distribution function *P(r)* was carried out using GNOM (version 5.0) (56, 57).

$$P\left(r\right)=\frac{1}{2{\text{π}}^{2}}\overset{\infty }{\underset{0}{\int }}\text{I}\left(Q\right)Qr\;\sin\left(Qr\right)\text{d}Q$$

*P(r)* corresponds to the distribution of distances *r* between the volume elements in the macromolecule. This yields the maximum dimension of the macromolecule *L* and its most commonly occurring distance vector *M* in real space. For this *P(r)* analysis, the X-ray *I(Q)* curve utilized up to 788 data points in the *Q* range between 0.09 and 1.50 nm^−1^ for both glycosylated and deglycosylated IgG3. The neutron *P(r)* curve utilized up to 134 *I(Q)* data points in the *Q* range between 0.165 and 1.60 nm^−1^ for both glycosylated and deglycosylated IgG3.

### Atomistic modeling of IgG3

Starting structures were created for each of glycosylated and deglycosylated IgG3 based on the sequence of Figure 2. The Fab structure (Fig. 1) was based on the chimeric IgG4 b72.3 crystal structure (PDB ID: 1BBJ) (43), and the Fc structure was based on the serum-derived IgG3 antibody Fc crystal structure (PDB ID: 5W38) (58). Modeller Version 9.19 (57) was used to generate the full-length IgG3 structure by substituting the A33 Fab sequence into the chimeric IgG4 b72.3 structure. The IgG3 hinge region was built using a PyMOL script build_seq (PyMOL Script Repository, Queen's University), based on the ^216^ELKTPLGDTTHTCPRCP(EPKSCDTPPPCPRCP)_3_APELLGGP^285^ sequence.

This 62-residue hinge structure was subjected to energy minimization and molecular dynamics (MD) independently of the Fab and the Fc regions in order to identify hinge conformations. Hinge structures were prepared for all-atom molecular dynamics simulations using Glycan Reader and Modeler (59, 60, 61) at the CHARMM-GUI website (http://www.charmm-gui.org/). Protonable residues were identified using the PDB2PQR website (http://apbs-rest-test.westus2.cloudapp.azure.com/pdb2pqr) and their protonated states adjusted for pH 6 in CHARMM-GUI. The TIP3P model was used to simulate explicit water molecules. The octahedral solvation box was used as this allowed for at least 1.0 nm from the protein in each axis, and 0.138 M NaCl was added. The CHARMM36 force field was used (62, 63), and all calculations were performed at 293.1 K. The particle mesh Ewald algorithm was applied to calculate electrostatic forces, and the van der Waals interactions were smoothly switched off at 1.0 nm by a force-switching function (64). A time step of 2 fs was used in all simulations. Initially, the system was shortly equilibrated in constant particle number, volume, and temperature (NVT) condition using CHARMM36 (65). To assure gradual equilibration of the system, positional restraints for backbone and side-chain heavy atoms were applied and the restraint forces were gradually reduced during the equilibration. The system was simulated for 100 ns and repeated three times using the CHARMM36 force field on the Kathleen high-performance cluster at University College London using NAMD (66). For the production, particle number, pressure, and temperature (NPT) simulation, the Langevin coupling coefficient was set to 1 ps^−1^ and a Nosé-Hoover Langevin-piston (67, 68) was used to maintain constant pressure (1 bar) with a piston period of 50 fs and a piston decay of 25 fs. The time step was 2 fs, and trajectories were saved every 100 ps. The electrostatic interactions were updated every 20 fs. The short-range nonbonded and electrostatic interactions were calculated with a cutoff of 1.2 nm. SHAKE was used to constrain all bonds involving hydrogen atoms. Convergence of all repeats was checked through the comparison of the average RMSD using VMD (69).

A PCA of the frames from all three repeat simulations was used to identify commonly occurring hinge conformations. Five such hinge conformations were used to build five full-length IgG3 starting structures by attaching the above structures for the Fab and Fc regions. This approach of creating several start structures using theoretical hinge conformations minimized a potential bias if a single hinge conformation had been used to create full-length IgG3 structures. To attach the Fab regions to the hinge, bonds were created using PyMOL between the mainchain atom of Val215 (the last residue of the Fab region) and that of Pro285 (the first residue in the hinge region). To attach the Fc region to the hinge, which was not straightforward, MD simulations of the hinge included a small segment ^239^SVFLFPPK^246^ (EU numbering) in the IgG3-Fc crystal structure that was kept unchanged in the MD simulations while allowing the hinge to move. This ^239^SVFLFPPK^246^ peptide was the anchor site for superimposing the hinge to the Fc region. The two N-linked Asn297 glycans on the Fc C_H_2 domains were approximated as complex-type biantennary oligosaccharides with a Man_3_GlcNAc_2_ core and two NeuNAc.Gal.GlcNAc antennae (12). The glycan template for this was taken from the GitHub repository (https://github.com/dww100), which was energy minimized using NAMD (70) for 1 ns to achieve a relaxed structure. This glycan was added to the Fc region by bringing its C1 atom in its first GlcNAc residue to within 0.14 nm of the Asn297 sidechain N atom in the C_H_2 domain, while ensuring no steric clashes with the Fc residues and the glycan chain. The PDB file was then opened on Discovery Studio (Dassault Systèmes BIOVIA) in order to create “CONECT” records for these glycosidic bonds. The CHARMM force field parameters and protein structure file, including those for the disulfide bridges and glycans, were generated using the CHARMM-Gui GlycanReader tool (70, 71, 72) in order to be compatible with the CHARMM36 forcefield (63, 73, 74, 75, 76). This procedure was followed for all five starting structures. To relax all ten starting structures (five with and five without glycans), these were energy minimized using the simulation engine NAMD version 2.9 with the CHARMM36 forcefield.

For the Monte Carlo simulations to generate trial structures, the residues in the five starting IgG3 structures were renumbered and their naming nomenclature was adjusted to match the required format for the Torsion Angle Monte Carlo (TAMC) module on SASSIE-web for this to work (77). The IgG3 residue numbering was thus changed into one continuous segment that encompassed both the Fab and the Fc regions. A library of physically realistic glycosylated and deglycosylated structural conformations was generated by subjecting the five starting hinge structures to the TAMC module in SASSIE-web (77). The peptides that were conformationally varied were assigned within the hinge. For glycosylated IgG3, these peptides were ^217^LKTP^220^ and ^285^PS^286^ on one hinge of IgG3, and ^217^LKTPLG^222^ on the other hinge (Figs. 1*B* and 2*E*). For deglycosylated IgG3 these peptides were ^217^LKT^219^ and ^283^ GG^284^ on one hinge, and ^217^LKTP^220^ on the other hinge of IgG3. These six peptides corresponded to surface-accessible structures outside the structurally defined Fab and Fc regions and the disulfide-linked hinge core. These peptides were structurally varied in TAMC to create the required IgG3 conformers for testing against the scattering curve, while the rest of the IgG3 model was held rigid. Allowing ^217^LKTP^220^, ^217^LKTPLG^222^, or ^217^LKT^219^ to be variable on both IgG3 hinges enabled both Fab regions to be conformationally mobile. Allowing ^285^PS^286^ or ^283^ GG^284^ to be variable enabled the Fc region to be mobile too. For each of these linker residues, the backbone phi (ϕ) and psi (ψ) torsion angles were varied in 15^o^ steps. During the Monte Carlo simulations, many attempted steps were physically unrealistic and were therefore discarded by TAMC. Overall, for glycosylated IgG3, 50,000 moves were attempted for each of the five starting hinge structures. Of these, 31% to 61% were rejected because of inappropriate steric clashes to leave a total of 135,135 of 250,000 starting models that were accepted. For deglycosylated IgG3, in which the glycan chains were omitted, 250,000 moves were attempted, of which 57% to 85% of the models were rejected to leave 73,905 models that were accepted.

For the two libraries of 135,135 and 73,905 models, a scattering curve was generated for each model using the SasCalc module in SASSIE-web. SasCalc calculated the scattering curve *I(Q)* using an all-atom expression for the scattering intensity in which the orientations of the *Q* vectors are taken from a quasi-uniform spherical grid generated by the golden ratio (31). For X-ray modeling, consideration of the hydration shell would require the explicit addition of a monolayer of water molecules to the protein surface before calculating *I(Q)* and would require much computational effort (31). Thus, the hydration shell was not considered here for X-rays and was not required for neutrons. These scattering curves were compared with the SAXS and SANS experimental curves extrapolated to zero concentration, using the R-factor function in SASSIE-web. This function calculates the difference between the modeled curve *I*_*Model*_*(Q*_*i*_*)* and the interpolated experimental curves *I*_*Expt*_*(Q*_*i*_*)*, this function being analogous to that used in protein crystallography:

$$R=\;\frac{\sum I_{Expt}\left(Q_{i}\right)-\;\eta \;I_{Model}\left(Q_{i}\right)}{\sum I_{Expt}\left(Q_{i}\right)}\times \;100$$

where *Q*_*i*_ is the *Q* value of the *i*^th^ data point, *I*_*Expt*_*(Q*_*i*_*)* is the experimental scattering intensity, *I*_*Model*_ *(Q*_*i*_*)* is the theoretical modeled scattering intensity, and η is a scaling factor used to match the theoretical curve to the experimental *I(0)* (55). Lower R-factor values represent better fits. An iterative search to minimize the R-factor was used to determine η (55). The use of χ^2^ analyses to evaluate the fits was not possible because this requires the experimental data points to have errors associated with them, which were not available upon interpolation of the curve. In the extrapolated experimental scattering curves, the lowest *Q* values in the range before the fitted Guinier *R*_*G*_ region were interpolated to zero *Q* using MATLAB in order to satisfy the input requirement for the SasCalc module in SASSIE-web. Interpolation makes the *Q* spacing uniform between the data points, and extrapolation extends the full *I(Q)* curve to zero *Q*. The resulting 825 and 146 *I(Q)* values in the *Q* range of 0.0 to 1.5 nm^−1^ were utilized for the SAXS and SANS curve fits, respectively, and defined the *Q* spacing for use in SasCalc and the R-factors. The use of χ^2^ analyses to evaluate the fits was not possible because this requires the experimental data points to have errors associated with them, which were not available when interpolating the curve. For the SANS curve fits, a correction of 2.0% of *I(0)* was required to allow for a flat incoherent background that was attributed to the proton content of IgG3 and the heavy water dialysis buffer (78). The 135,135 glycosylated and 73,905 deglycosylated models gave an R-factor *versus R*_*G*_ distribution that encompassed the experimental extrapolated *R*_*G*_ value. This R-factor analysis was repeated for four experimental SAXS curves at different concentrations for each of glycosylated and deglycosylated IgG3 (Table 2). The same analysis was repeated for two SANS curves at different concentrations for each of glycosylated and deglycosylated IgG3 (Table 3). For each concentration, the best-fit 100 models with the smallest R-factors were accepted. All the accepted models were subjected to short energy minimizations to correct any broken bonds that may have arisen during the TAMC simulation. All the calculated parameters in Tables 2 and 3 were calculated both prior to and after the energy minimization to monitor its effect on the extracted structures. None of the parameters underwent a significant change after energy minimization. Moreover, two best fit curves (one before and one after energy minimization) are shown in Figures 11 and 12 to demonstrate that the short energy minimizations had a negligible effect on the theoretical SAXS and SANS curves.

PCA provided by the Bio3d package in R (79) was used to identify the main classes of best-fit IgG3 conformations found in the 400 best-fit glycosylated and deglycosylated SAXS models (Table 2). A separate analysis of the 400 best-fit SANS models was performed. To remove any bias in the PCA clustering of coordinate sets caused by the presence or absence of the glycans, the glycans were removed from the best-fit glycosylated models prior to generating the PCA. The midpoint structure for each PCA group was identified using a centroid model computed using R. For reference, the best-fit structures for glycosylated and deglycosylated IgG3 at 1.00 mg/ml, respectively, are available in supporting information.

In order to model AUC parameters the theoretical *s*_*20,w*_ values were generated for the best-fit 800 glycosylated and deglycosylated SAXS and SANS IgG3 models using HullRad (29) (Tables 2 and 3). Hullrad includes glycan residues for glycosylation; however, there are inconsistencies in the Protein Database nomenclature for glycans. The nomenclature in the HullRad script was thus modified to ensure that the IgG3 glycosylation was correctly incorporated in the *s*_*20,w*_ calculation.

## Data availability

All data are contained within this article. The two single best-fit models for glycosylated and deglycosylated IgG3 from X-ray searches at 1.00 mg/ml (Fig. 12) are available in supporting information. These best-fit glycosylated and deglycosylated IgG3 structures were also deposited in the SASDBD database (https://www.sasbdb.org/) with reference codes SASDLZ2 and SASDL23.

## Supporting information

This article contains supporting information.

## Conflict of interest

The authors declare that they have no conflicts of interest with the contents of this article.
